# Carbamoyl phosphate and its substitutes for the uracil synthesis in origins of life scenarios

**DOI:** 10.1038/s41598-021-98747-6

**Published:** 2021-09-29

**Authors:** Louis M. P. Ter-Ovanessian, Baptiste Rigaud, Alberto Mezzetti, Jean-François Lambert, Marie-Christine Maurel

**Affiliations:** 1 Laboratoire de Réactivité de Surface (LRS, UMR 7197 CNRS), Case courrier 178, Sorbonne Université, 4, Place Jussieu, 75005 Paris, France; 2 Institut de Systématique, Evolution, Biodiversité (ISYEB), Muséum National d’Histoire Naturelle, Sorbonne Université, Ecole Pratique des Hautes Etudes, Université des Antilles, CNRS, CP 50, 57 rue Cuvier, 75005 Paris, France; 3 Sorbonne Université, Institut des Matériaux de Paris Centre (FR2482), 4 Place Jussieu, 75005 Paris, France

**Keywords:** Biogeochemistry, Astrobiology, Origin of life

## Abstract

The first step of pyrimidine synthesis along the orotate pathway is studied to test the hypothesis of geochemical continuity of protometabolic pathways at the origins of life. Carbamoyl phosphate (CP) is the first high-energy building block that intervenes in the in vivo synthesis of the uracil ring of UMP. Thus, the likelihood of its occurrence in prebiotic conditions is investigated herein. The evolution of carbamoyl phosphate in water and in ammonia aqueous solutions without enzymes was characterised using ATR-IR, ^31^P and ^13^C spectroscopies. Carbamoyl phosphate initially appears stable in water at ambient conditions before transforming to cyanate and carbamate/hydrogenocarbonate species within a matter of hours. Cyanate, less labile than CP, remains a potential carbamoylating agent. In the presence of ammonia, CP decomposition occurs more rapidly and generates urea. We conclude that CP is not a likely prebiotic reagent by itself. Alternatively, cyanate and urea may be more promising substitutes for CP, because they are both “energy-rich” (high free enthalpy molecules in aqueous solutions) and kinetically inert regarding hydrolysis. Energy-rich inorganic molecules such as trimetaphosphate or phosphoramidates were also explored for their suitability as sources of carbamoyl phosphate. Although these species did not generate CP or other carbamoylating agents, they exhibited energy transduction, specifically the formation of high-energy P–N bonds. Future efforts should aim to evaluate the role of carbamoylating agents in aspartate carbamoylation, which is the following reaction in the orotate pathway.

## Introduction

Understanding the origins of nucleotides is not only essential for understanding the origins of life (OoL) in the frame of the “RNA world” hypothesis, but also boasts relevance to pharmaceutical applications^1^. This topic belongs to the realm of protometabolism, a term referring to a primitive metabolism^2^ in living organisms and, by extension, to metabolic-like pathways related to the origin of life. The idea of an evolution of biosynthetic pathways progressively evolving from prebiotic chemistry dates back to the works of Horowitz in 1945^3^. He proposed that molecules which are products of present-day metabolic pathways might have been nutrients, directly harvested from the primordial soup. Later on, when this supply of free chemicals was exhausted, the primitive organism had to evolve from heterotrophy towards autotrophy using self-made metabolites, constructed from simpler precursors. As a consequence, metabolites appearing at the first steps of a given pathway would be the last ones to have evolved.

After Horowitz’s work, abiotic analogues of current metabolic pathways were proposed. Cairns-Smith explored in his book the transmission and reproduction of information based on features of clay minerals, a concept called “genetic takeover”^4–6^. Wächtershäuser laid the foundations of a systematic approach aiming to predicting surface-based analogues of metabolic pathways^7,8^, where mineral surfaces played the role of heterogeneous catalysts which would later be taken over by enzyme catalysts. Many of his suggestions still remained untested.

In recent years, the idea of an intimate, reciprocal relation between the mineral and organic worlds has been the object of renewed interest^9,10^. Recent work of the Sutherland group on energy transfers for nucleotides and protometabolic precursors highlighted how prebiotic chemistry must be closely intertwined with geochemistry and climatic parameters^11–13^. The protometabolic approach aims to discover molecular permanence of the past. By exploring inorganic reaction networks, which were analogous to extant metabolic pathways, this approach follows a principle of continuity. Core aspects of metabolism would not need to have been created from scratch, since they would be elaborations of pre-existent abiotic reactions. It also satisfies the principle of parsimony (Ockham’s razor) and it is experimentally testable, and thus falsifiable, allowing progress “from conjecture to hypothesis”^14^. Traces of this hybrid world might be found in activated molecules, between which energy transduction mechanisms are carried out.

**Figure 1:**
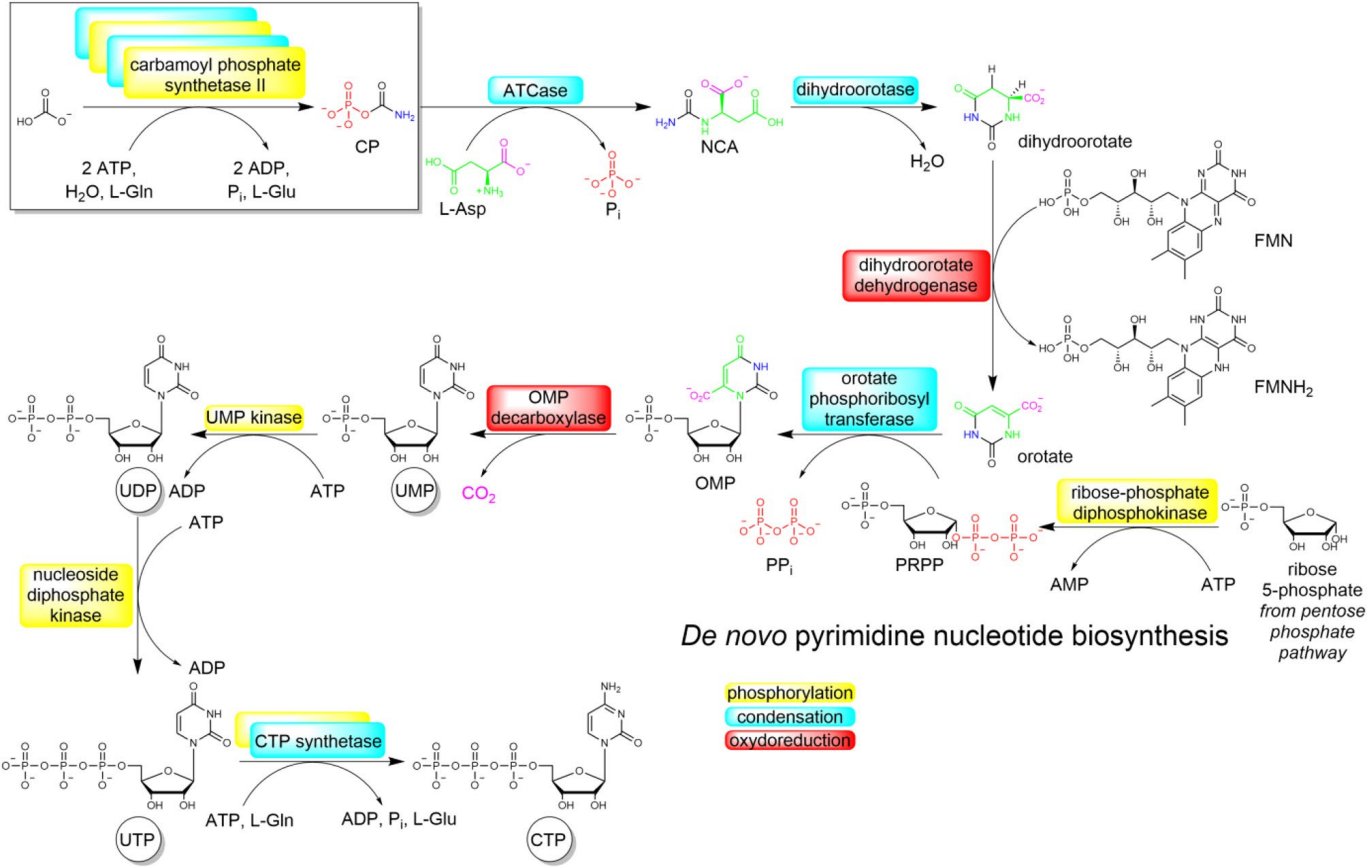
Current in vivo pyrimidine biosynthesis along the orotate pathway. Catalysing enzymes are indicated in boxes.

This work is focussed on the first steps of the pyrimidine nucleotide synthesis, specifically uridine-5’-monophosphate (UMP). The core ring, leading ultimately to a nitrogenous base, is formed first during UMP biosynthesis (Fig. 1). The sugar moiety, D-ribose issued from phosphoribosyl pyrophosphate (PRPP) is then incorporated to the structure^15^.

**Scheme 1:**
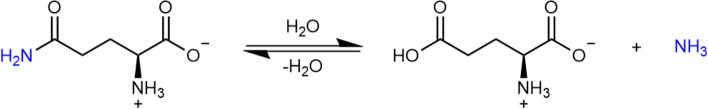
Decomposition of glutamine, releasing ammonia.

The pyrimidine ring atoms come from L-aspartic acid (L-Asp) and carbamoyl phosphate (CP). Formation of the latter molecule is the first step of the pathway and is catalysed by an enzyme named carbamoyl phosphate synthetase II. This enzyme uses glutamine (L-Gln) as a nitrogen source (Scheme 1).

CP is also a key species in the urea cycle, which occurs within mitochondria. In this cycle, carbamoyl synthetase I directly uses ammonia as a nitrogen source for synthesising CP.

**Scheme 2:**
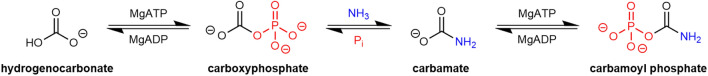
Carbamoyl phosphate enzymatic formation steps.

Carbamoyl synthetase features three active sites connected to a molecular channel^16^. This structures facilitates the successive performance of three chemical steps, starting from the hydrogenocarbonate ion that is derived from dissolved carbon dioxide (Scheme 2).

First, a mixed anhydride is formed. The phosphate source is adenosine triphosphate (ATP) (Scheme 2), leading to the carboxyphosphate ion. Second, aqueous ammonia produced in situ from glutamine decomposition substitutes the phosphate group of carboxyphosphate, yielding a carbamate ion. Finally, the carbamate is phosphorylated by ATP, generating CP^17^.

In the frame of the geochemical continuity hypothesis^10^, a transposition from the biochemical into the inorganic world may be considered. The question that needs to be asked is whether carbamoyl phosphate, the first high-energy compound in the biosynthetic pathway, is a potential prebiotic reagent, stable ex vivo in water, and/or in aqueous ammonia. Ammonia is a logical candidate to replace glutamine decomposition occurring in vivo. It is also the nucleophile yielding phosphoramidates (also know as amidophosphates)^18^ and conversely, it may also result from their decomposition. Finally, urea thermal decomposition^19^ as well as salmiac^20^ dissolution are plausible precursors for ammonia solutions (for a complete discussion see^21–23^).

In vivo, phosphate groups are added by a free-energy-rich molecule, ATP^24^. Prebiotic robust phosphorylating analogues, that are rich in energy, include trimetaphosphate ions, coming from volcanoes^25,26^, or phosphoramidates^27^. Their ammonolysis products can be substituted to ATP, as ATP is a far too complex assembly to have existed on the primordial Earth.

## Results

### Carbamoyl phosphate hydrolysis

A 0.355 M carbamoyl phosphate (CP) solution was prepared in D_2_O (D_2_O/CP = 155) and its evolution was followed. First, the ^31^P NMR spectrum was recorded shortly after dissolution (8 min, Fig. S1 in Supporting Information file). At this point, the solution still mostly contained unhydrolyzed CP (− 1.41 ppm^28^). A minority signal between + 2.74 and + 2.69 ppm (Q^0^ region) can be assigned to monophosphate ions that could derive from the hydrolysis of CP. Another complex shape between − 6.43 and − 6.66 ppm (Q^1^ region) and a signal at about − 20.12 ppm (Q^2^ region) could be due to linear triphosphate, probably an impurity. The complex shapes observed are due to initial pH drift as the solution was not buffered (Fig. S2). This is the only reaction for which significant pH drifts were observed; extrapolating from the first observed spectrum, the pH immediately after dissolution was 9.9.

**Scheme 3:**
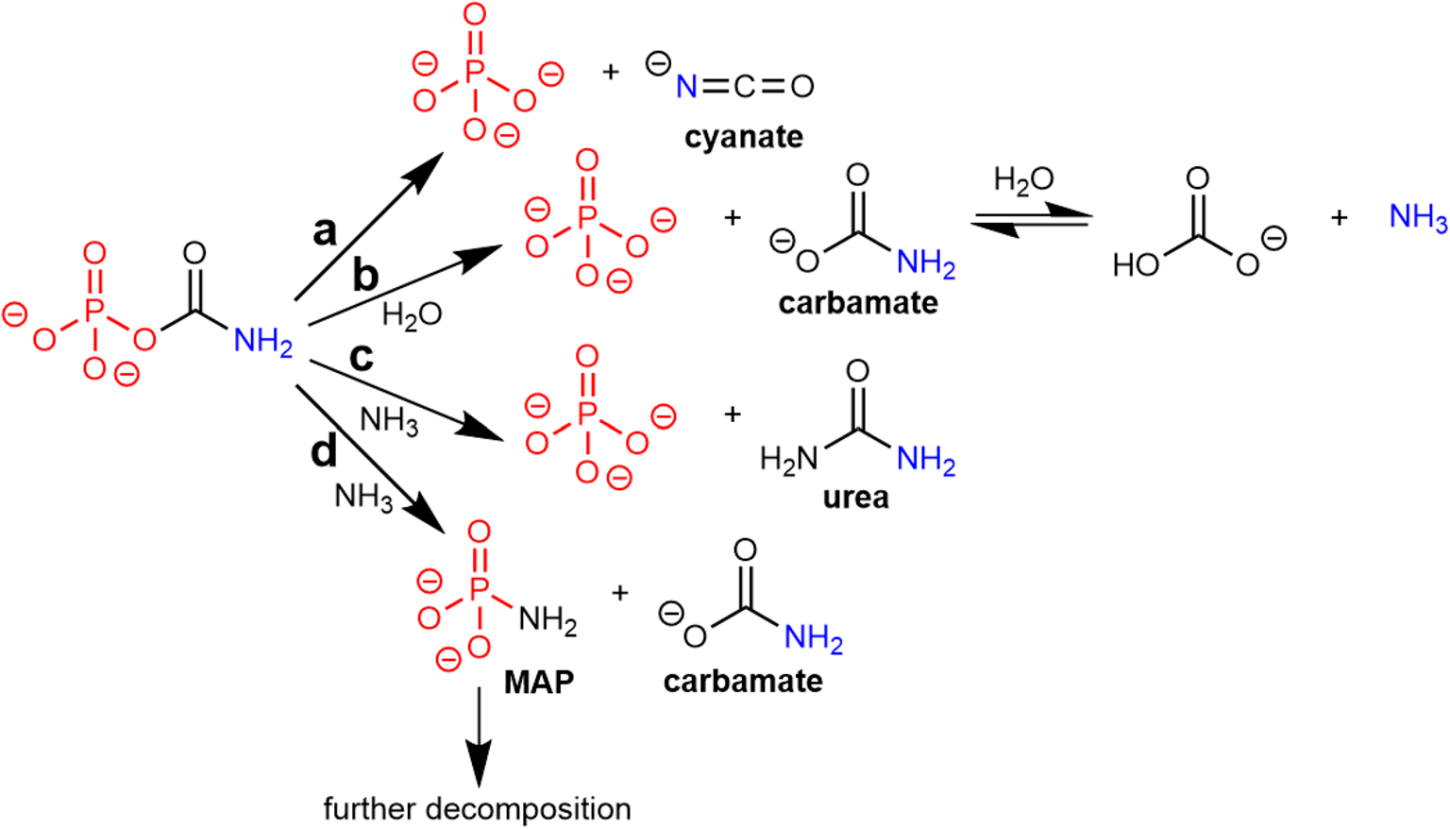
CP hydrolysis (**a**,**b**) and ammonolysis (**c**,**d**) reactions.

After 17 and 72 h evolution, ^31^P NMR spectra were also run (Figs. S2 and S5). CP decomposition proceeded to 92% after 72 h. Decomposition does not seem to follow first-order kinetics, however, the limited number of data points was not sufficient to fully characterize the kinetics. ^13^C NMR spectra (Figs. S3 and S4) were also recorded. Due to the long acquisition time, the spectra correspond to an average over 17 h of evolution. The signal of CP is found at the expected position of 157.4 ppm^29^, and accounts for about 36% of total ^13^C intensity. This value is coherent with the evolution of hydrolysis that could be estimated from the ^31^P results, taking into account that it was averaged over the accumulation time. The signal at 161.1 ppm can be assigned to a carbonate formed by complete CP hydrolysis^30^. Another signal at 129.0 ppm indicates the presence of the cyanate ion^31^, confirmed by ATR-IR spectroscopy (anti-symmetric stretching band at 2171 cm^−1^, Fig. S6). All NMR data can be rationalized by the reaction mechanism shown in Scheme 3 (paths a and b), where CP is subject to two decomposition paths with comparable kinetics. One path involves decomposition into cyanate and P_i_; the second path occurs through a hydrolysis of the P–O–C bond, yielding carbamate and formation of one P_i_.

Based on these results, a more complete kinetic study of the same 0.355 M CP solution in water was conducted using ATR-IR spectroscopy. This technique permits observation of cyanate species^32,33^ (ν_CN_ at 2168 cm^−1^, Fig. S7a) and phosphates, whether they are free or bonded (to carbamoyl moieties)^34,35^ (ν_PO_ around 1100 cm ^−1^, Fig. S6). The cyanate band was followed and quantified over 19 h, with a sampling frequency of one spectrum per hour. A calibration curve for cyanate was measured to model the kinetics of cyanate evolution (Fig. S7b,c). After an initial growth period, the cyanate concentration reaches a maximum of about 0.08 M (i.e. about 25% of the starting CP) at about 8 h, and then begins to slowly decrease. The initial cyanate formation can be described by a first-order kinetics, as expected from unimolecular decomposition of CP, with a t_1/2_ of ~ 150 min. Later on, at least one slow reaction decomposes cyanate. Most likely, it is hydration to carbamate, which is in equilibrium with hydrogenocarbonate.

In parallel, we followed the evolution of CP and free monophosphate by ^31^P NMR spectroscopy (peaks at − 1.55 and + 1.91 ppm, respectively) for 119 h (Fig. S8). The sum of the two ^31^P signals maintained a constant value, confirming that CP quantitatively decays to unbound phosphate. The CP evolution curve can be described by the sum of two kinetics profiles, one completed in a few hours, and the second one in a few days. Equilibrium is obviously not reached after 119 h for the slow process. The faster process corresponds to the unimolecular decomposition to cyanate and Pi (path a in Scheme 3), as the rate constant is of the same order of magnitude as the one determined by ATR-IR spectroscopy. The slower process is most likely hydrolysis to carbamate and Pi. This conclusion is corroborated by the observation that when the fast process is all but over, the reaction quotient of CP → carbamate decomposition is close to 1, corresponding to a Δ_r_G° close to zero. This is to be compared with the weakly negative value of − 2.85 kJ mol^−1^ reported at 303 K by Jones et al.^36^. First-order models give a relatively good fit of NMR data, although the pH is far from being constant in our experimental conditions. In fact, the data of Allen^37^ indicate that the rate constants are strongly pH-dependent only above pH 9, i.e. for the first points of Fig. S8. This pH dependency accounts for the difference between the values quoted here and those of Allen, and of Vieyra^38^ and Oestreich^39^.

### Carbamoyl phosphate ammonolysis

A 0.473 M CP solution was prepared in partly deuterated water containing 4.22 M ammonia (NH_3_/CP molar ratio = 8.94, H_2_O/CP = about 110, pH = 11.2). Its evolution was followed according to the same acquisition protocol as above (Figs. S9–S11) in a constant volume cell so that the ammonia content was constant too. The degradation of CP was very fast initially, with only 22% intact CP remaining even in the first ^31^P spectrum. The majority ^31^P signal (76%) was at + 3.48 ppm, corresponding to fully deprotonated monophosphate; an additional signal at + 8.45 ppm accounting for 2.6% of the total ^31^P intensity can be assigned to phosphomonoamidate (MAP)^27,40^.

At the end of the reaction period, CP had completely disappeared, while MAP and monophosphate were present approximately in the same ratio as in the first spectrum. Meanwhile, the ^13^C signal, averaged over 22 h evolution time, showed the signal of cyanate (majority, over 80% of total), carbonate, carbamate (respectively 166.6 and 168.1 ppm, Fig. S10), and additionally a peak at 163.8 ppm that is attributed to urea^41,42^.

Thus, carbamoyl phosphate in an ammonia-containing environment is subject to a more complex evolution than in water. In addition to the two decomposition pathways mentioned above, two addition-eliminations involving NH_3_ are possible (paths c and d in Scheme 3); either the phosphate group is attacked by NH_3_ and MAP is released, or the carbonyl group is attacked and phosphate is released while urea is formed.

**Figure 2:**
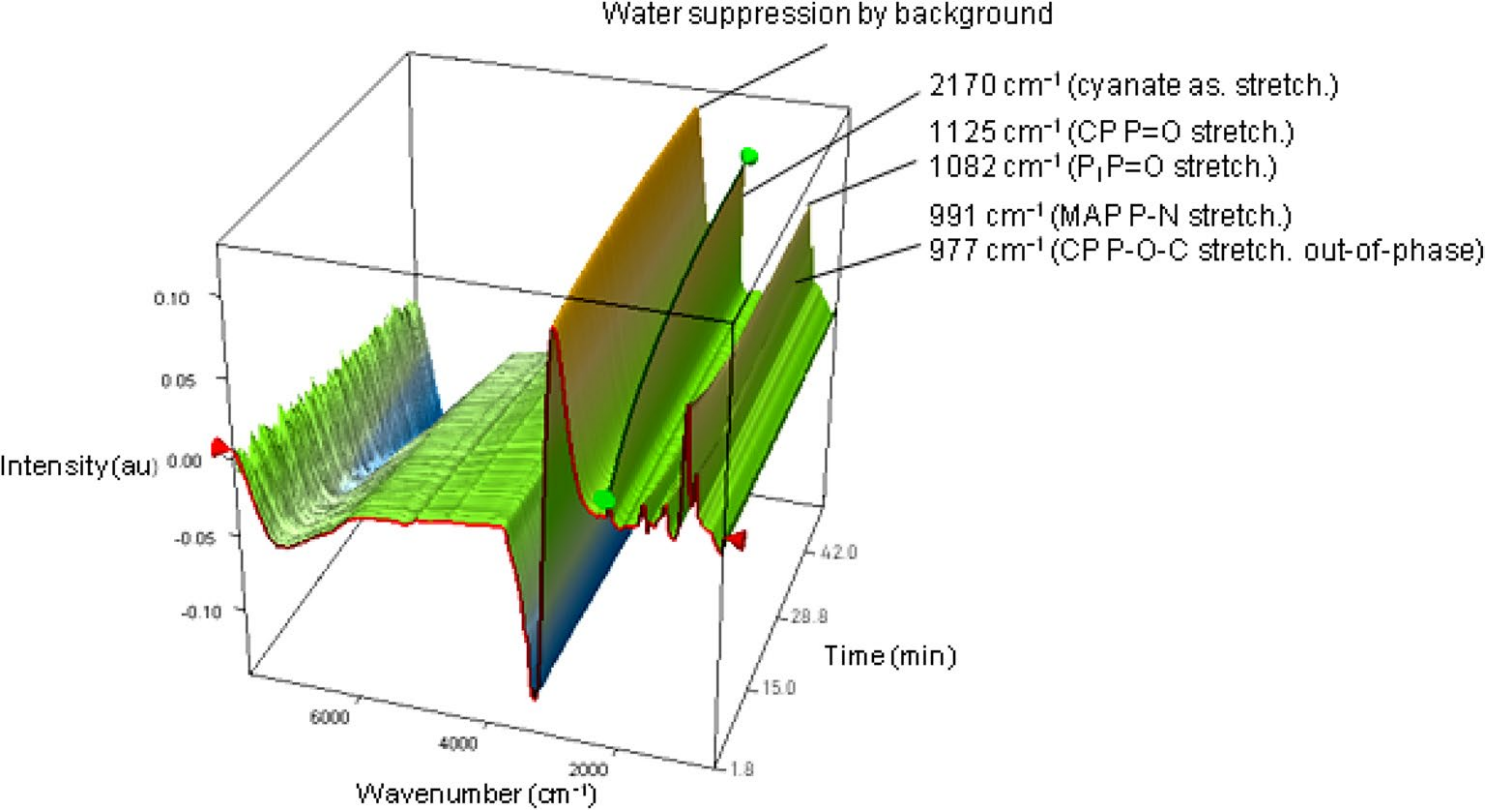
Stacked ATR-IR spectra of the CP kinetics in ammonia. The green highlighted curve corresponds to the evolution of the cyanate band.

A more complete kinetic study of the evolution in NH_3_/H_2_O was implemented by ultrafast ATR-IR spectroscopy. The cyanate vibration band at 2168 cm^−1^ was followed over 45 min, sampling one spectrum every 16 s (Fig. 2 and Fig. S12).

The observed t_1/2_ of cyanate formation is only 7.25 min, about 20 times faster than in D_2_O. Even though cyanate formation is formally an internal decomposition of CP, it appears to be subject to basic catalysis since it is much faster in the ammonia basic solution. The amount of cyanate reaches a plateau at 0.24 M.

In parallel, reaction kinetics was followed by ^31^P NMR spectroscopy, acquiring one spectrum every 12 min for 119 h. The three previously identified species were followed as a function of time (Figs. S13 and S14). Their evolution could be fitted with first-order kinetic rate constants of 7.92 × 10^–2^ min^−1^ (t_1/2_ = 8.72 min) for CP decomposition, 7.94 × 10^–2^ min^−1^ (t_1/2_ = 8.73 min) for P_i_ formation and 5.52 min^−1^ (t_1/2_ = 6.62 min) for MAP formation. The position of the monophosphate ^31^P peak was used as an internal pH-meter; it indicated that the pH drifted from 11.7 to 11.3 in the course of the reaction, considerably less than for the previous case of CP hydrolysis. All of the models are, in this case, consistent with Allen and Oestreich’s previous studies^37,39^ in the same range of basic pH (Fig. S15). In conformity with a remark of Vieyra^38^, we observed that adding an excess of ammonia drives the system into first order pathways.

Decomposition of CP was almost complete after ten minutes. This reaction proceeded through three parallel pathways occurring at similar rates: nucleophilic attack by NH_3_ to give MAP (limited to approximately 3% of initial CP), internal decomposition to cyanate (~ 51%) and hydrolysis to carbamate/carbonate (~ 46%). At this point, ~ 3% of the original activated molecules are transformed to a weaker phosphorylating agent, ~ 50% to a carbamoylating agent, and the rest is lost as a free energy source.

**Scheme 4:**
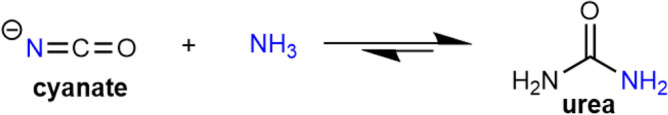
Urea formation after production of carbamate in an ammonia CP solution (Wöhler’s synthesis).

Final equilibrium is not reached yet at this point. ^13^C NMR spectra showed changes from the 40th to the 179th hour of reaction (Fig. S16). The quantities of carbamate and carbonate seemed constant (or, at least, did not show a clear change). Together, they account for one third to one half of the total intensity (compatible with IR quantification). Meanwhile, the amount of cyanate decreased and the amount of urea increased at approximately the same rate. This trend confirms that cyanate is consumed by reaction with NH_3_, producing urea (Wöhler synthesis^43^, Scheme 4). Urea can still be seen as a carbamoylating agent, but it is likely to be a less reactive one than cyanate since it appears thermodynamically favoured in these conditions.

### Trimetaphosphate as a free energy source

#### Trimetaphosphate (P3m) hydrolysis

**Scheme 5:**
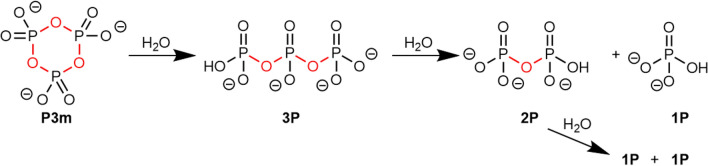
The NHP (natural hydrolysis products) of trimetaphosphate. High free energy P–O–P bonds are outlined in red.

Previous experiments show that carbamoyl phosphate is fairly unstable in aqueous solution. Another logical aim was to find a way to (re)generate it using a source of free energy—presumably another “activated” molecule that could be injected into the medium. In order to explore this reactivity, sodium trimetaphosphate is a possible alternative; it can be generated by both volcanic eruptions^25^ and condensation of phosphates^44,45^. Furthermore, sodium trimetaphosphate has already been shown to produce phosphorylation in the presence of various reagents^46–48^. It contains three phosphoric anhydride bonds (P–O–P) that may be considered as “high-energy bonds” in aqueous solution, meaning that their hydrolysis is exergonic. The natural hydrolysis products (NHP)^49,50^ would include mono-, di- and triphosphate anions, as illustrated in Scheme 5.

To check trimetaphosphate stability, a P3m solution in water (0.488 M) was prepared and analysed by ^31^P NMR spectroscopy at the initial time and after 45 days’ maturation at 25 °C (Fig. S17). The signal of the P3m species is visible on the ^31^P NMR spectrum as an intense singlet at − 21.10 ppm. After 45 days, the major constituent of the solution is still P3m (at − 21.19 ppm^47^, 99.0% of the total phosphorus amount), and mono- and triphosphates are present in trace amounts. P3m in its native form is therefore metastable, over tens or even hundreds of days at ambient temperature.

#### Trimetaphosphate ammonolysis

**Scheme 6:**
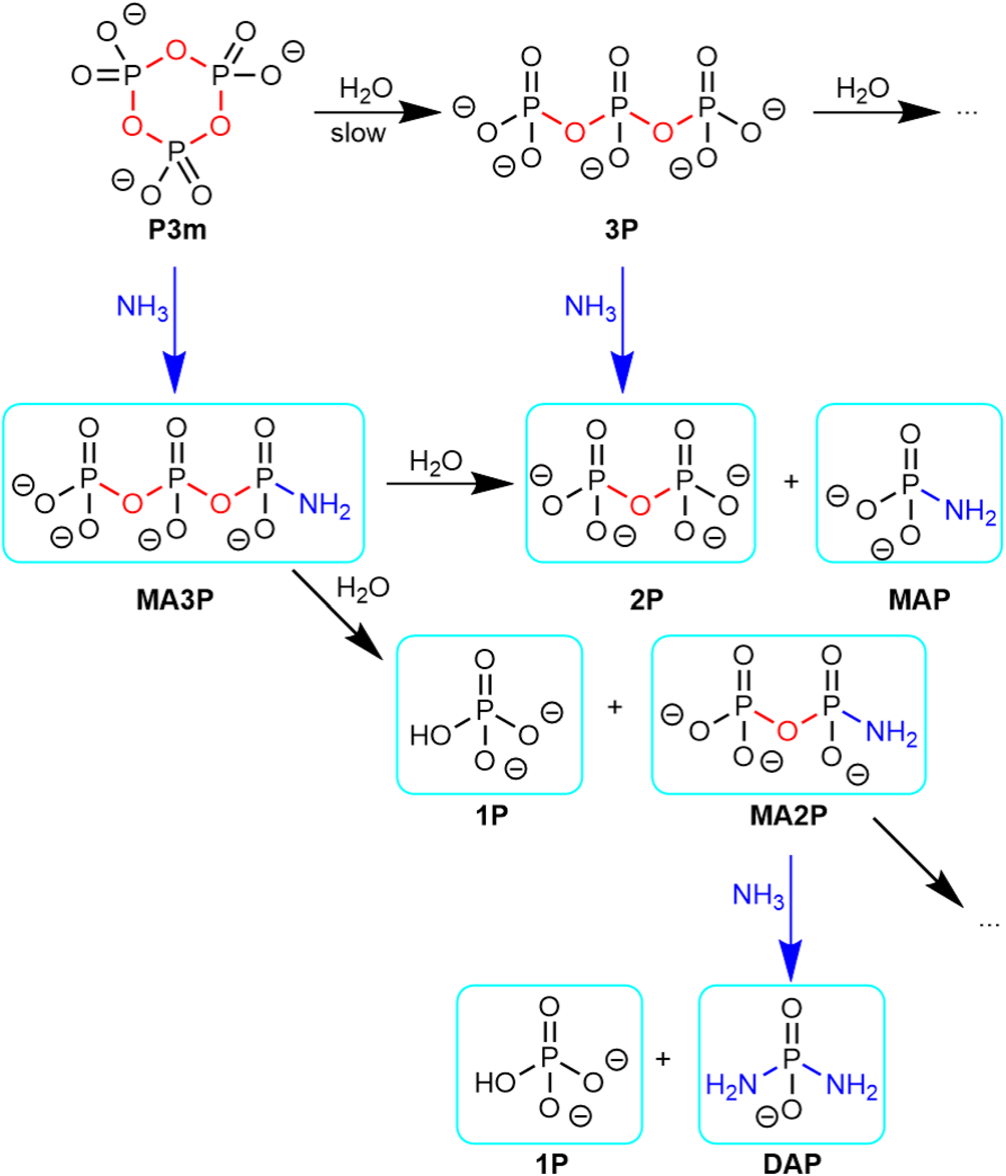
The NAP (natural ammonolysis products) of trimetaphosphate (only some of the possible reactions are shown). “High free energy” bonds are outlined in red (P–O–P) or in green (P–N). Species effectively observed by NMR spectroscopy are circled in blue.

P3m is known to produce more reactive phosphorylating agents when reacted with ammonia. Based on the original protocols of Quimby^51^ and Feldmann^52^, P3m was left to age in the presence of concentrated NH_3_, and analysed using ^31^P NMR spectroscopy. A solution of 2.5 g of sodium trimetaphosphate in 8.3 mL of 28% ammonia and 16.7 mL of water (P3m concentration = 0.327 M, NH_3_/P3m molar ratio = 250) was prepared and placed in a tightly closed bottle in an oven at 70 °C for 66 h. These conditions are similar to hydrothermal OoL scenarios^53^. After 66 h, pH decreased from 11.79 to 11.3 (room temperature). The ^31^P NMR spectrum showed: MAP (0.9% of total P), DAP (5.6%), MA2P (42.6%), MA3P (13.0%), 1P (23.1%), 2P (14.5%; Fig. S18). The first four compounds are phosphoramidates, containing a P–N link. The formation of phosphoramidates was confirmed by ATR-IR spectroscopy (Fig. S19), showing phosphoramidate bands^34^ at 1192 cm^−1^ (P=O stretch.), 1095/1082/1013 cm^−1^ (P–N/P–O stretch.), 990 cm^−1^ (P–N stretch.), and 932 cm^−1^ (P–N stretch.). They may be called “NAP” (natural ammonolysis products, Scheme 6). The hydrolysis products were not directly detected, even at high temperature (70 °C).

The solution obtained after this treatment contains a large variety of species resulting from the opening of the P3m cycle. Most of them still possess P–O–P ad/or P–N bonds, and the solution may therefore be considered as a “high free energy” solution. It is, in fact, still far from chemical equilibrium, although it does not spontaneously decay at measurable rates at low temperatures. To test these hypotheses, the solution evolution was first monitored at 30 °C, ^31^P NMR spectroscopy showed no detectable change of speciation for 2 h. The solution was then aged for 30 days at 100 °C in a tightly closed bottle in an oven, conditions possibly harsh enough to lead to chemical equilibrium. Indeed, the ^31^P spectrum after harsh activation showed full hydrolysis to monophosphate, part of which precipitated as ammonium phosphate, identified by NMR spectroscopy after redissolution. The various compounds present in the solution obtained at 66 °C can definitely be qualified as “high-free energy”^18^ as the equilibrium state is so strongly displaced towards hydrolysis.

We have not attempted to fully characterize the interconversion of NAP (and NHP) species in the course of ageing. The first step is the opening of P3m to MA3P, already rapid at 30 °C. Kinetic monitoring performed for 42 h at 30 °C (Figs. S20 and S21) shows that the concentration of P3m falls to undetectable levels following simple first order kinetics (rate constant k = 9.2.10^–3^ min^−1^). The three signals assigned to M3P increase at the same rate.

When this intermediate step is completed, one of the three P–O–P (phosphoric anhydride) bonds has been replaced by one P–NH_2_ (phosphoramidate) bond in M3P. This reaction is overwhelmingly displaced to the right. Thus, the phosphoramidate bond, while still “high-free energy”, stores less free energy than the original phosphoric anhydride bond. However, the M3P molecule obtained is more labile and therefore constitutes a more efficient phosphorylating agent than P3m. Many successful phosphorylations have been reported using MA3P^54^.

While speciation does not evolve beyond MA3P at a perceptible rate at 30 °C, the solution aged at 66 °C shows five other products that are outlined in Scheme 6. We find among them the phosphodiamidate (DAP), which is among the most studied phosphorylating agents. Krishnamurthy et al. showed the versatility of this molecule, which is even more effective than the MA3P^55^ from which it can be synthesized, in terms of prebiotic phosphorylation^56,57^.

### Can trimetaphosphate generate carbamoylating agents?

An open question remains if P3m and/or the phosphoramidates derived from it are able to phosphorylate carbamoyl donors, yielding CP analogues.

#### P3m + ammonium carbamate

**Scheme 7:**
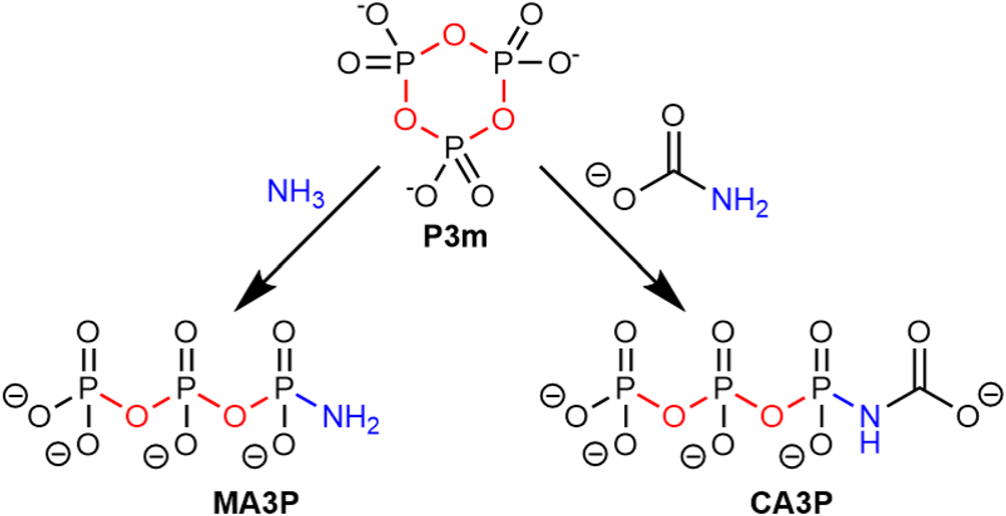
P3m evolution in the presence of ammonium carbamate.

The reactivity of P3m with ammonium carbamate was investigated first. An aqueous solution of sodium trimetaphosphate (0.33 M) and ammonium carbamate (2.97 M, carbamate/P3m molar ratio = 9) was prepared. After 3 h of reaction at room temperature, ^31^P NMR spectrum (Fig. S22) revealed traces of pyrophosphates (− 6.60 ppm)^58^ and monophosphate (NHP). The pH drift over the course of the experiment was − 0.16. P3m was still present, but only accounted for about one quarter of the total intensity. In addition, signals are observable in three different regions with comparable intensities: − 0.36 to − 0.50 ppm, − 6.10 to − 6.25 ppm, and − 21.0 to − 21.35 ppm. Composite shapes are visible in each region. In fact, it seems that the solution contains two similar species, each with three non-equivalent P atoms in an approximate 3:1 ratio. One of these species is probably MA3P^27^, although the peaks are shifted with respect to those that result from P3m ammonolysis, undoubtedly due to the pH difference (9.48 instead of 10.45)^59,60^. The other species is probably the MA3P derivative shown in Scheme 7, namely *N*-carbamoyl triphosphate (*N*-CA3P) in which the P atoms would indeed have chemical environments similar to MA3P.

After one week at 25 °C (Fig. S23), small quantities (< 1%) of MAP and DAP species^27^ are formed, possibly due to the slow hydrolysis of MA3P. MA3P then accounts for 97.3% of the total phosphorus, and N-CA3P is no longer present. The amide bond of N-CA3P is likely to be highly susceptible to hydrolysis, as it is in carbamate, and hydrolysis at this position would result in conversion of N-CA3P to MA3P.

A new signal series also appears: − 6.02 (singlet), − 12.11 (doublet) and − 20.67 (triplet) ppm. In total, these contribute 0.67% to the total amount of phosphorus. The signal likely originates from a triphosphate derivative, other than MA3P.

A ^13^C NMR spectrum was also acquired after one week of ageing (Fig. S24). Only two signals were observed, one corresponding to the carbamate/carbonate equilibrium pair and the other to urea. This is not unlike the final product of CP ammonolysis. In particular, no cyanate is present, confirming that this species lies at a high free energy position, inaccessible from carbamate.

In summary, the reaction of carbamate with P3m can yield a significant amount of a potential phosphorylating and carbamoylating agent (CA3P). However, the latter has a lifetime inferior to a few days, after which length of time the solution resembles the product of carbamate ammonolysis.

#### P3m + urea

We next replaced carbamate by urea. Since a mixture of these two compounds hardly evolves at room temperature, we applied thermal activation. An aqueous solution of sodium trimetaphosphate (0.33 M) and urea (1.18 M; urea/P3m molar ratio = 3.6) was prepared. Activating for 70 h at 70 °C caused a pH shift from 6.5 to 7.9. ^31^P NMR analysis (Fig. S25) did not show any reaction products between P3m and urea. Only mono-, di- and triphosphates were formed as products of natural hydrolysis^49^, accounting for about 9% of total ^31^P intensity.

Ammonium carbamate was then added to the previous (P3m + urea) solution, reaching an ammonium carbamate concentration of 11.9 M (carbamate: urea molar ratio = 10:1). After 1.5 day ageing at room temperature, ^31^P NMR spectroscopy (Fig. S26) showed the formation of MA3P (59% of total P). After 3.5 days (Fig. S27), the main signals were identical to the previous analysis, but MA3P has evolved to 73% of total intensity. A minority triplet signal also became apparent in the Q^2^ region (1 to 2% of total intensity), suggesting a triphosphate derivative that could be different from MA3P and N-CA3P judging from its chemical shift; perhaps a triphosphate containing a P–O–C link (P–O–CO–OH or P–O–CO–NH_2_). In summary, urea did not show any significant nucleophilic properties that would allow opening the P3m cycle, although its presence did not inhibit the action of other nucleophiles such as NH_3_ (and possibly carbamate).

An ATR-IR analysis on the solution supplemented the data from ^31^P NMR spectroscopy (Figs. S28 and S29). Bands at 1018 and 990 cm^−1^ were probably due to two types of P–N stretching (compatible with the formation of MA3P), while the small band at 1041 cm^−1^ could be due to a P–O–C stretching^34,61^, supporting the existence of minority carboxyphosphates. By comparison with a urea reference (Fig. S29), it appears that initial urea was consumed, while the carbamate/hydrogenocarbonate species are still present^62,63^.

#### P3m + ammonium hydrogenocarbonate

Next, ammonium carbonate was tested for trimetaphosphate opening. An aqueous solution of sodium trimetaphosphate (0.33 M) and ammonium carbonate (2.97 M, close to saturation, NH_3_/P3m ratio = 18, hydogenocarbonate/P3m = 9, pH = 8.2) was prepared and aged at room temperature. After 2 h, the ^31^P NMR signals (Fig. S30) were broad, perhaps due to the high viscosity of the solution near its saturation point. The main observed signal was that of unreacted P3m. The broad shoulder might originate from MA3P but could not be accurately quantified. After 2 days, the pH was 8.05 and the ^31^P NMR spectrum (Fig. S31) still showed P3m as the majority species (about 62%), but this time the signals of MA3P were clearly apparent.

ATR-IR analysis (Fig. S31) confirmed the presence at this point of hydrogenocarbonate (1613, 1358 cm^−1^), free ammonium (1450 cm^−1^) dissolved CO_2_ (2344 cm^−1^) and P3m (1650, 1267, 1088, 1000, 774 cm^−1^)^64,65^. The small band at 924 cm^−1^ is probably attributable to P–N symmetric stretching, supporting the presence of phosphates. No band could be assigned to P–O–C moieties.

After 4 days, the ^31^P NMR (Fig. S32) and ATR-IR (Fig. S34) spectra show a further decrease of P3m signals (to 38% of total). MA3P signals became predominant on the NMR spectrum, correlated with the P–N IR signature (924 cm^−1^). The minority signal already observed in the P3m + urea series was also present (about 2% of total). ^13^C NMR analysis (Fig. S35) reveals the presence of both carbamate and hydrogenocarbonate. The carbamate/hydrogenocarbonate ratio corresponds to approximately 3%, compared to 4% calculated from published equilibrium constants^30^ (neglecting the amount of NH_3_ immobilized by the formation of MA3P).

In summary, the reaction pattern of P3m with ammonium (hydrogeno)carbonate is similar to that with ammonium carbamate. This is not surprising since the latter two species are in equilibrium with each other. However, ammonium (hydrogeno)carbonate solutions contain less free NH_3_ because of their lower pH, and thus the reaction is significantly slower.

### Reactivity of phosphoramidate-containing solutions

#### Phosphoramidates + urea

Urea had been found unreactive when contacted with P3m, so we tested its reactivity with respect to a solution containing phosphoramidates, which are considered to be better phosphorylating agents^66^ than trimetaphosphate. Urea (0.102 g, 1.7 mmol) was dissolved into 2.5 mL of the phosphoramidates solution (obtained by P3m ammonolysis, cf. § 3 above). After 70 h reacting time at room temperature, ^31^P NMR spectrum (Fig. S36) showed no significant change compared to the initial phosphorylating solution. On the associated ^13^C NMR spectra (Fig. S37), the only signal observed belonged to urea at 163.7 ppm. The ATR-IR spectrum (Fig. S38) of the (phosphoramidates + urea) solution was simply the superimposition of the individual spectra of phosphoramidates solution and of urea solution.

#### Phosphoramidates + ammonium carbamate

A direct phosphorylation of ammonium carbamate was also attempted at 25 °C using similar reaction conditions. Ammonium carbamate (0.1347 g, 1.7 mmol) was dissolved into 2.5 mL of an phosphoramidate solution. After incubating 70 h at room temperature, ^31^P NMR analysis (Fig. S39) showed no significant difference compared to the initial phosphorylating solution. In particular, there was no evidence of CP, which could be expected to form from the active phosphorylating agents present in solution.

## Discussion

**Figure 3:**
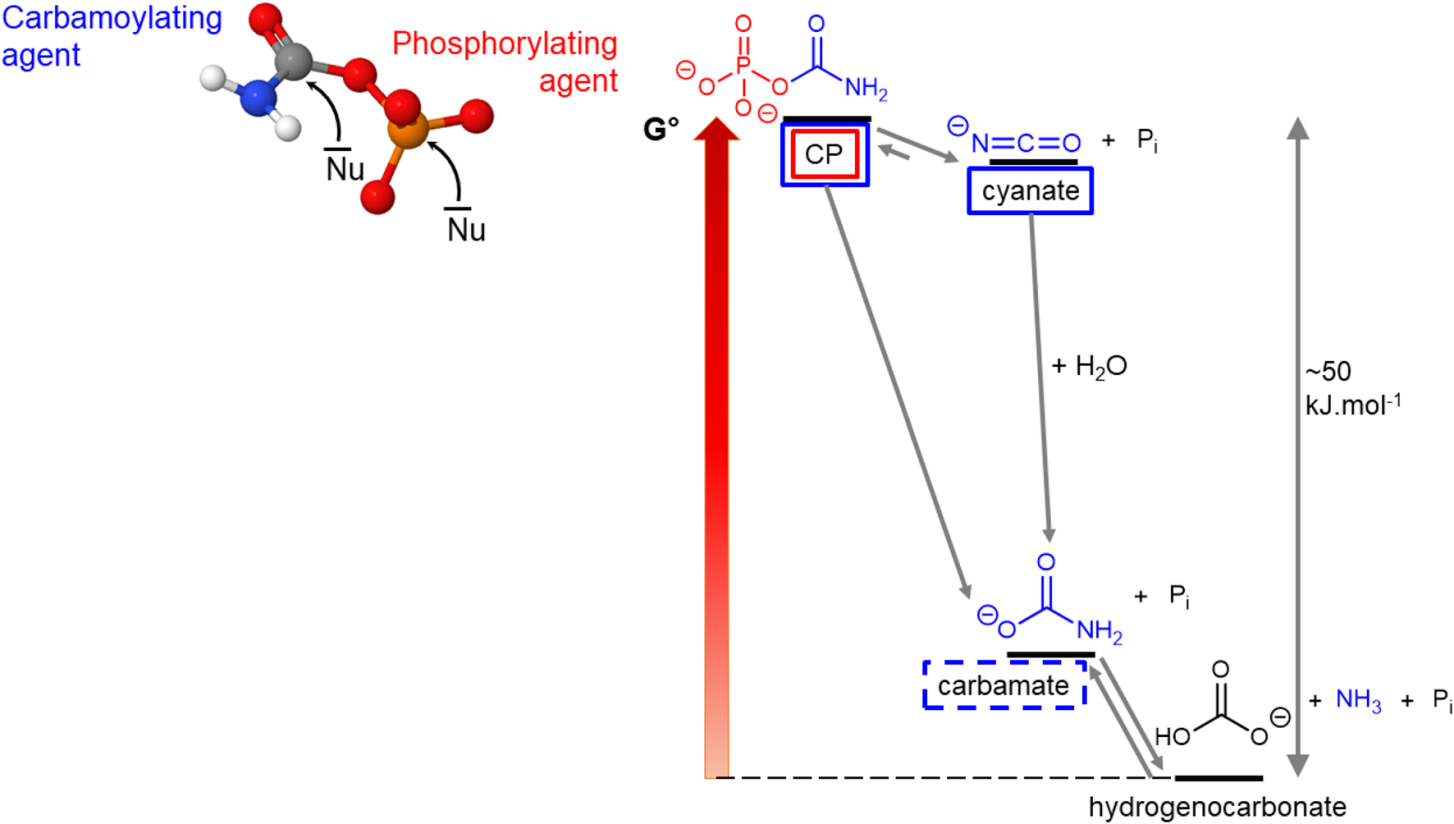
An energy diagram of CP degradation reactions in water at natural pH. CP itself could act as a carbamoylating agent (boxed in blue) or a phosphorylating agent (boxed in red). When it is isomerised to cyanate + P_i_, only the carbamoylating power is kept. Carbamate (^−^O_2_C–NH_2_) still contains the O–N–C moiety, but its carbamoylating power is weak due to its low free enthalpy.

Carbamoyl phosphate, a key compound for the energetic metabolism in the cell, is not stable in aqueous solution at room temperature, where it is ultimately decomposed into ammonia, hydrogenocarbonate and free phosphate. The sequence of transformations is summarized in Fig. 3. In aqueous solution, CP can be considered as an activated molecule, a reservoir of free enthalpy because it will ultimately degrade through a reaction that has a strongly negative Δ_r_G°. It is tempting to consider that the high free energy is exclusively due to the phosphoric ester (C-O-P) bond as it is true of many phosphorylated biomolecules. However, this is misleading, as most of the free enthalpy content is conserved after loss of the phosphate to give cyanate. Cyanate, however, is able to make use of its free enthalpy through carbamoylation, not through phosphorylation, while CP had a “double ability”. The fact that cyanate + P_i_ is close in enthalpy to CP is corroborated by the fact that the reverse reaction is a well-known method for generating carbamoyl phosphate^36,67,68^.

**Scheme 8:**
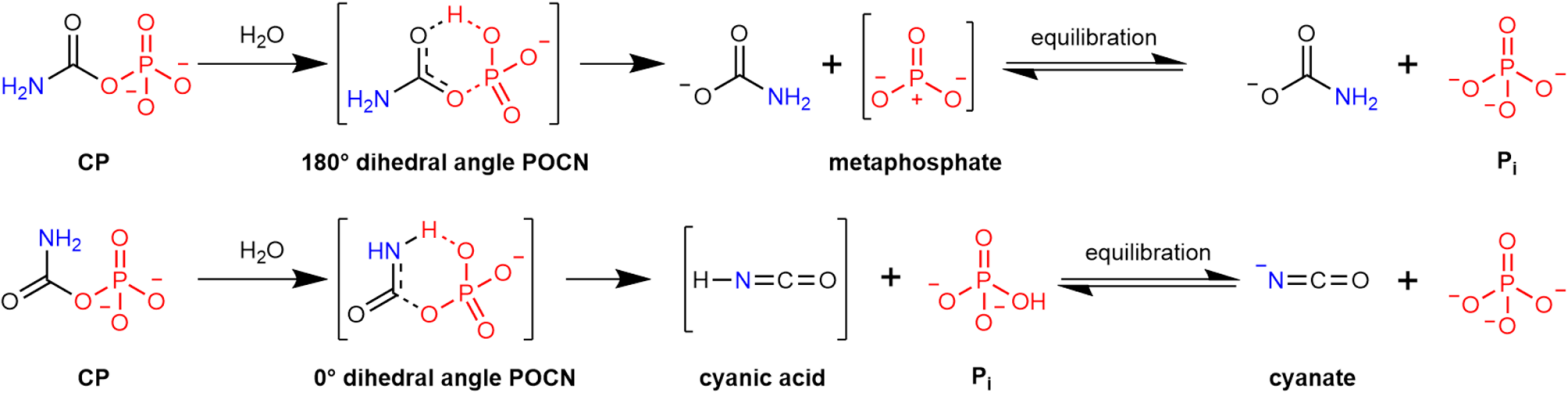
Enzymatic decomposition mechanisms of carbamoyl phosphate according to POCN dihedral angle^69^.

In water, the cyanate and the carbamate decomposition pathway proceed in parallel at comparable rates. In enzymes, Wang et al.^69^ have established that transcarbamoylases can orient the decomposition pathway depending on the POCN dihedral angle (Scheme 8). In the living cell, they orient the nucleophiles to the carbonyl while other enzymes target the phosphorus centre instead^70^.

**Figure 4:**
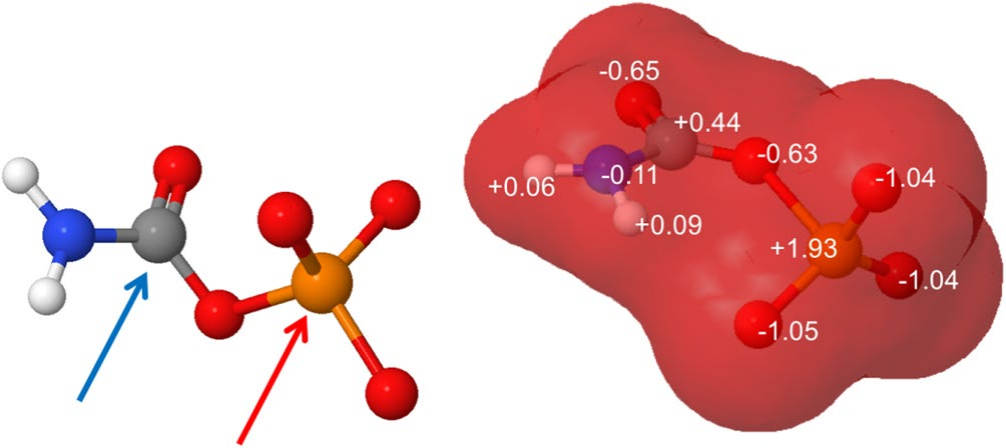
(**a**) Potential electrophilic sites on carbamoyl phosphate (**b**) Calculated charge distribution on carbamoyl phosphate.

In aqueous media of high ammonia concentration, CP could undergo two concurrent ammonolysis reactions in addition to the main decomposition reaction observed in pure water. In the first one, the ammonia nucleophile would attack the phosphorus centre, leading to MAP and carbonate. In the second, it would attack the carbon centre, leading to urea and monophosphate (Fig. 4a).

Experimentally, urea is the thermodynamically favoured product in the conditions we used. Yet it does not form at a measurable rate directly from CP. In contrast, MAP formation, which is the result of a nucleophilic attack on the P atom, is very fast. Modelling with MolCalc^71^ indicates that the partial charge on the carbonyl is + 0.44 while that of phosphorus is + 1.92 (Fig. 4b). As a consequence, kinetically, phosphorus is more reactive in spite of the fact that partial charges on neighbouring atoms (oxygen atoms of the phosphate group) could create a more repulsive shell than the flat carbonyl.

Thus, in high NH_3_ concentration conditions at room temperature, CP is transformed to several carbamoyl donors (urea, cyanate) and a phosphorylating agent (MAP). They could be positioned on a free enthalpy diagram resembling that of Fig. 3. Respective levels would be affected by the pH conditions in the NH_3_-rich solution (pH 11.3), with the carbamate/carbonate equilibrium favouring carbamate and the cyanate/urea favouring urea. From a kinetic point of view, cyanate remains available for a timescale of a few days at room temperature before decaying. Urea remains available for longer periods.

Let us consider the implications for an abiotic realization of the first steps of the orotate pathway (Fig. 1). Provided aspartate is present in the system, an “activating agent” is needed to carbamoylate it. From a thermodynamical point of view, this agent should be able to provide enough free enthalpy to overcome the unfavourable amide bond formation. This would exclude free carbamate, but includes CP, cyanate, and probably urea. The kinetic requirements are even more stringent. The proposed agent should be kinetically inert (metastable) in water, so that it is not degraded as soon as it is formed. Researchers often state that prebiotic reagents should be “stable” in solution. This term is misleading because “stability” normally refers to a thermodynamic feature, while the question here is a kinetic one.

In the presence of aspartate, there should exist a reaction mechanism that allows transferring free enthalpy to amide bond formation instead of letting it go to waste. This transfer should also be significantly faster than degradation in water. On this count, CP itself, which decomposes in a few hours at room temperature, is less promising than cyanate or urea, which are both stable for several days in the same conditions.

The same combined thermodynamic and kinetic approach can be applied to solution containing high-enthalpy phosphorylating agents. P3m is a “high-energy” molecule as the hydrolysis of its three P–O–P bonds can release a substantial amount of free enthalpy, but it is kinetically inert (metastable for months at room temperature).

**Figure 5:**
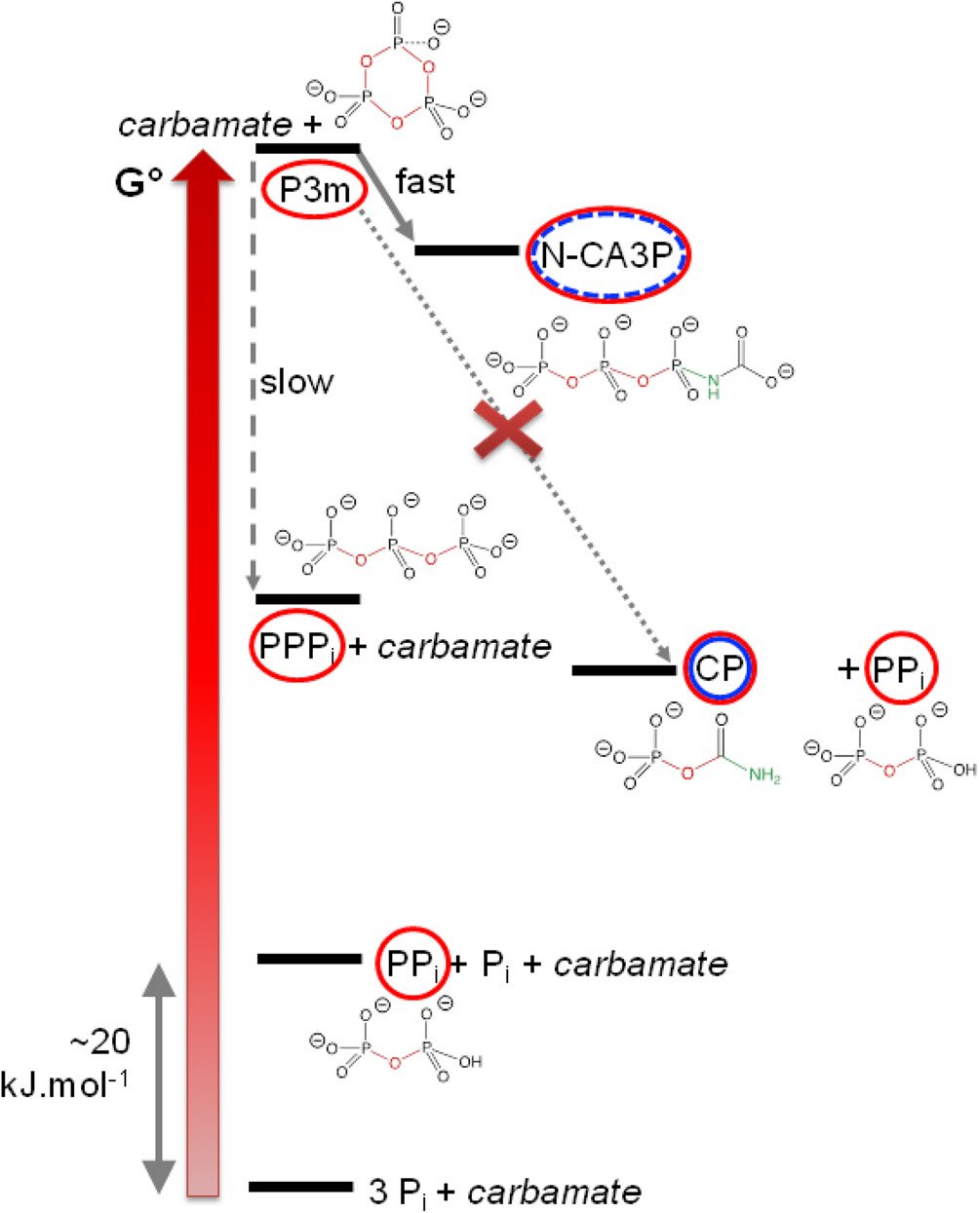
A partial energetic view of P3m degradation reactions in aqueous solutions containing carbamate. Thermodynamic data from Pasek^24^ have been used.

Contact with an ammonia-containing solution quickly generates a slightly more stable triphosphate (MA3P), followed by other species of successively lower free enthalpy. Figure 5 represent some (observed and unattested) reactions of P3m in a carbamate-containing solution. The positions on the free enthalpy scale are approximate, based on thermodynamic data compiled by Pasek^24^.

Hypothetically, reaction of these two species could yield CP (together with diphosphates) and thus provide a prebiotic source of the latter. In fact, this reaction is not observed. The hypothesized N-CA3P species were formed in significant amounts (about 20% of ^31^P signal intensity after 3 h) although, from a mechanistic point of view, they appear unable to act as a carbamoylating agent. A nucleophilic attack by the Asp –NH_2_ would result in the formation of a carboxyl, not a carbamoyl group. In the results section, an O-carbamoyl triphosphate was mentioned too. Even though this minority species suits better for carbamoylation, it was not fully identified and, at any rate, it represents less than 1–2% of NMR signal intensity. It is still arguable that it could be a carboxyphosphate. Finally, the long-term product of reaction (not represented in Fig. 4) is MA3P, which does not contain the carbamoyl moiety and forms preferentially from the amount of free NH_3_ that is always present in carbamate solutions.

Reaction with urea, theoretically also able to provide activated carbamoylating agents, did not occur at a measurable rate even when heating the system to 70 °C. Even using the activated phosphoramidate solution was not efficient in this respect, although it contained species such as DAP that were demonstrated by Krishnamurthy et al. to constitute efficient phosphorylating reagents.

Finally, one may wonder to what degree our conclusions are dependent on the pH of the solution. While in modern microorganisms the pH is kept constant by homeostatic mechanisms, the latter did not exist in prebiotic environments, although some conditions, such as the presence of mineral surfaces, might have led to local pH buffering. Our choice here was to use unbuffered solutions as more representative of the spontaneous evolution of prebiotic systems. The drawback is that reaction rate measurements cannot lead to accurate values of rate constants. However, in most systems under study, the pH drift remained limited, and in the case where a strong pH drift was observed (CP hydrolysis), the effect over reaction rates were arguably not important. Therefore, we suggest that our main conclusions are valid in a broader range of conditions than those studied in the present work.

## Conclusions

Our research program aiming to check the likelihood, in non-biological conditions, of successive steps of the orotate pathway, we studied the probability of CP presence in prebiotic aqueous solutions and its ability to act as a carbamoylating agent. ^31^P NMR spectroscopy was particularly suitable to identify the reaction products, supplemented by ATR-IR and ^13^C NMR spectroscopy.

Our findings tend to show that CP itself is not likely to have played a significant role in prebiotic chemistry. First, it is too labile and would decompose quickly if formed in solution. CP is a mixed anhydride (phosphoric acid and carbamic acid) and thus it is very sensitive to hydrolysis. Second, attempts to form CP using well-known phosphorylating agents (P3m and its ammonolysis products) were unsuccessful. Other paths for energy degradation were followed by the system.

These results do not definitely exclude CP as a prebiotic reagent. Aqueous solutions were the only environment considered herein. A different conclusion could be deduced from dry state chemistry (for example by using mineral surfaces in the absence of water). Other reactions conditions such as hydrothermal scenarios must also be explored. Interactions with mineral surfaces could modify the thermodynamics and kinetics of the observed reactions. However, in aqueous solutions at room temperature, two of the degradation products of CP, cyanate and (perhaps) urea, do constitute promising carbamoylating agents as they endure for many days in solution. Furthermore, they could be produced by other geochemical pathways^72,73^, not involving prior phosphorylation.

The next step in our research plan, which will be the object of a later publication, will explore the reactivity of these potential carbamoylating agents towards aspartate in solution and on mineral surfaces.

## Methods

The following chemical compounds were purchased from commercial suppliers and used without further purification: carbamoyl phosphate disodium salt (Sigma-Aldrich Co., cat. n°C4135-1G), ammonia 28% analaR Normapur (VWR Chemicals, cat n°21190.292), sodium cyanate (Sigma-Aldrich Co., cat. n°185086-100G), trisodium trimetaphosphate (Sigma-Aldrich Co., cat. n°T5508-500G), ammonium carbamate (Sigma-Aldrich Co., cat. n°292834-100G), urea ACS reagent (Sigma-Aldrich Co., cat. n°U5128-100G), ammonium carbonate ACS reagent (Aldrich chemical company, Inc., cat. n°20,786-1), deuterium oxide 99.90% D (Eurisotop, cat. n°D214FE).

### NMR experiments

NMR experiments were conducted on a Bruker Avance III 500 spectrometer (ω_L_ = 500.07 MHz for ^1^H, 202.43 MHz for ^31^P and 125.74 MHz for ^13^C) equipped with a 5 mm inverse double resonance broadband probe. Chemical shifts were calibrated as δ values (ppm) relative to the peak of TMS set at δ = 0.00 ppm (^13^C NMR), D_2_O set at δ = 4.79 ppm (^1^H NMR), 85% H_3_PO_4_ in water set at δ = 0.00 ppm (^31^P NMR). Coupling constants are given in Hertz. All spectra were processed in Bruker TopSpin 4.0.6 and 4.0.8. All kinetic studies were processed in Bruker Dynamics Center 2.5.4.

pH measurements were carried out using a Fischer Scientific Accumet AE150 pH Benchtop Meter.

The rationale for ^31^P signals assignments is discussed in the supplementary information.

### FTIR-ATR spectral measurements

FTIR measurements were carried out using a FTIR spectrometer (Bruker, VERTEX 80) equipped with a mono-reflection diamond ATR device (Bruker, A225/Q-DLST). The refractive index of the diamond is 2.4. The radiation from the IR source of the spectrometer was focused into the ATR crystal and the output radiation (from the other side of the crystal) was focused onto a nitrogen cooled MCT (Bruker, D316-L/B) detector. Measurements were carried out in the spectral range 650–7000 cm^−1^ under RapidScan mode, with a mirror speed of 160 kHz. Each spectrum was an average of 250 scans to increase the signal to noise ratio and the background (water) was an average of 1000 scans.

A medium layer of approximately 20 µL was placed on the diamond ATR crystal. An injection cell of 30 µL (Bruker, A220-FL) was used for avoiding evaporation in the case of kinetic measurements.

Using the kinetic measurements option in the OPUS software (Bruker, OPUS 7.8), we measured the spectra of the solutions (CP in water / CP in ammonia) every hour / 16 s.

### Preparation of phosphoramidate phosphorylating solution

2.523 g of P3m were dissolved into 16.7 mL distilled deuterated water and 8.3 mL 28%w/w ammonia. The solution was then heated in a sealed flask at 70 °C for 66 h into an oven. 600 µL were sampled for NMR analysis.

### MolCalc

The MolCalc web interface (molcalc.org) developed by J. H. Jensen and J. C. Kromann was used in order to simulate partial charges of carbamoyl phosphate.

## Supplementary Information

Supplementary Information.
