# Supplementary material for: Carbamoyl phosphate and its substitutes for the uracil synthesis in origins of life scenarios
Source: Sci Rep. 2021 Sep 29;11:19356. doi: 10.1038/s41598-021-98747-6 (PMC8481487; doi:10.1038/s41598-021-98747-6)
Supplement: Supplementary file 1 — Supplementary Information. [file 41598_2021_98747_MOESM1_ESM.docx]

Supplementary material for article

Carbamoyl phosphate and its substitutes for the uracil synthesis in origins of life scenarios

**Louis M. P. Ter-Ovanessian^1,2^, Baptiste Rigaud^3^, Alberto Mezzetti^1^, Jean-François Lambert^1^ (corresponding author) and Marie-Christine Maurel^2^**

*^1^ Laboratoire de Réactivité de Surface (LRS UMR 7197 CNRS), Sorbonne Université, CNRS, F-75005 Paris, France. jean-francois.lambert@sorbonne-universite.fr*

*^2^* *Institut de Systématique, Evolution, Biodiversité (ISYEB UMR 7205 CNRS), Muséum national d'Histoire naturelle, Sorbonne Université, Ecole Pratique des Hautes Etudes, Université des Antilles, CNRS ; CP 50, 57 rue Cuvier 75005 Paris, France. marie-christine.maurel@sorbonne-universite.fr.*

*^3^* *Institut des Matériaux de Paris Centre (FR 2482 CNRS), Case courrier 178, Sorbonne Université, 4, Place Jussieu, F-75005 Paris, France.*

Table of contents

Abbreviations and chemical nomenclature……………………………………………………SI 1

Results

1. Carbamoyl phosphate hydrolysis………………………………………………….SI 2
2. Carbamoyl phosphate ammonolysis………………………………………………SI 10
3. Trimetaphosphate stability in water or ammonia....................................................SI 15
4. Trimetaphosphate evolution in presence of carbonylated compounds...................SI 19
5. Phosphorylation attempts using phosphoramidates.................................................SI 32
6. Empirical table of encountered ^13^P chemical shifts……………………….……...SI 36

**Abbreviations and chemical nomenclature**

1P, P_i_: monophosphate, orthophosphate ion

2P, PP_i_: diphosphate ion

3P, PPP_i_: linear triphosphate ion

CP: carbamoyl phosphate

DAP: diamidophosphate ion

MA2P: monoamidodiphosphate ion

MA3P: monoamidotriphosphate ion

MAP: monoamidophosphate ion

P3m: cyclic trimetaphosphate ion

**Results**

- 1. Carbamoyl phosphate hydrolysis

D**euterated water + carbamoyl phosphate at 25°C**

NMR data:

^31^P NMR (Bruker, 202.43 MHz, D_2_O, 25°C, ppm), from 0 to 12 min

1P: δ 2.72 (s); Carbamoyl phosphate: -1.41 (s); -5.68 (m); 3P: -6.57 (m); 3P: -20.12 (m)

^13^C NMR (Bruker, 125.74 MHz, D_2_O, 25°C, ppm), from 12 min to 16h 58 min

Carbonate at pH 8.5: δ 161.09 (s); Carbamoyl phosphate: 157.37 (s); Cyanate: 129.54 (s)

^31^P NMR (Bruker, 202.43 MHz, D_2_O, 25°C, ppm), from 16h 58 min to 17h 10 min

1P: δ 1.74 (s); Carbamoyl phosphate: -1.78 (s); -6.79 (m); 2P: -7.44 (s)

^13^C NMR (Bruker, 125.74 MHz, D_2_O, 25°C, ppm) t_0_ + 1d 10h 52 min

Carbonate at pH 8.5: δ 161.08 (s); Carbamoyl phosphate: 157.39 (s); Cyanate: 129.43 (s)

^31^P NMR (Bruker, 202.43 MHz, D_2_O, 25°C, ppm), after 3 days

1P: δ 2.32 (s); Carbamoyl phosphate: -1.46 (s); 3P: -6.55 (d, 20Hz); 2P: -7.14 (s); 3P: -20.81 (t, 20Hz)

pH measure: 8.20 after 3 days

**
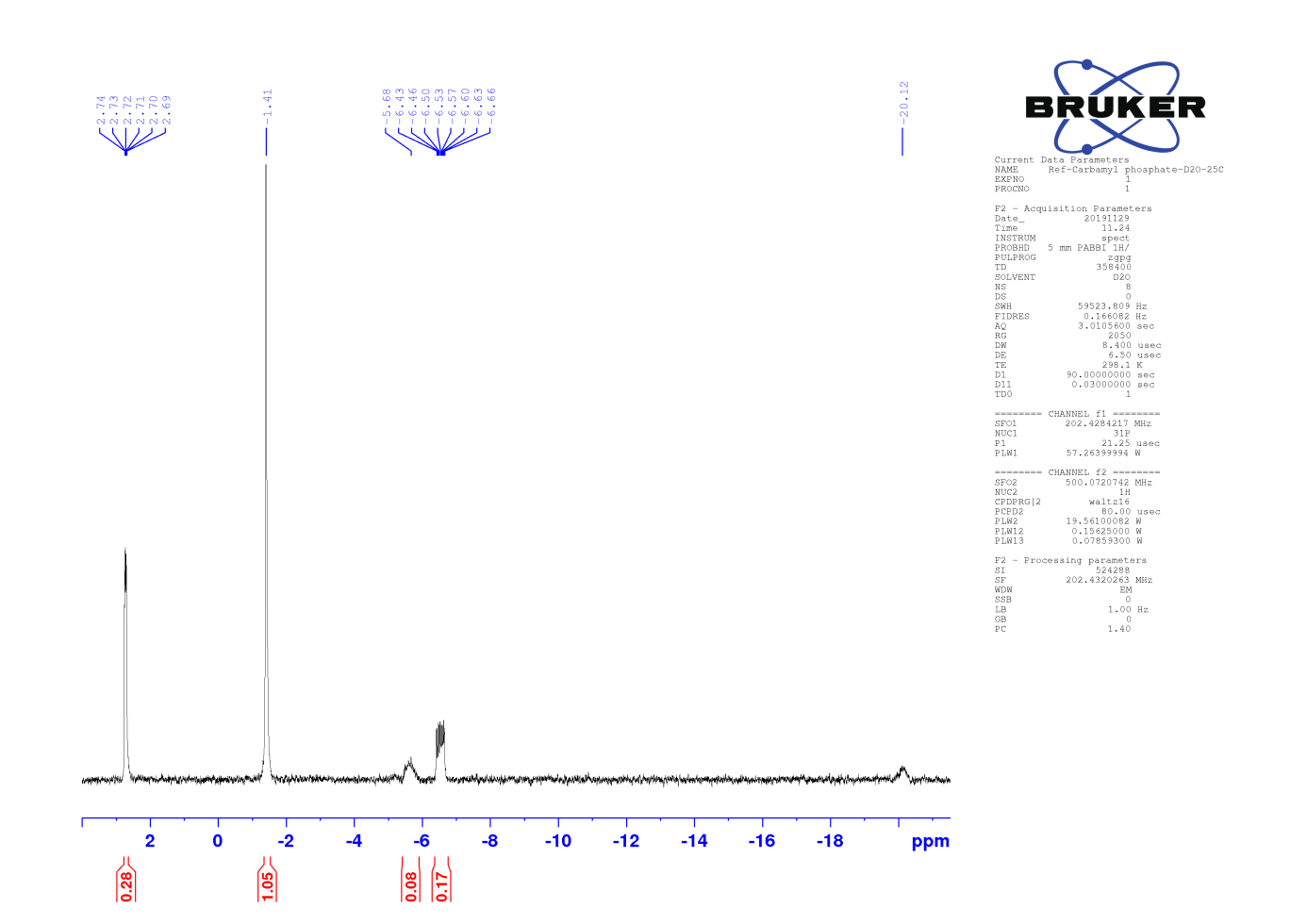
**

**Fig. S1** ^31^P NMR spectrum of carbamoyl phosphate in deuterated water at 25°C after 12 min


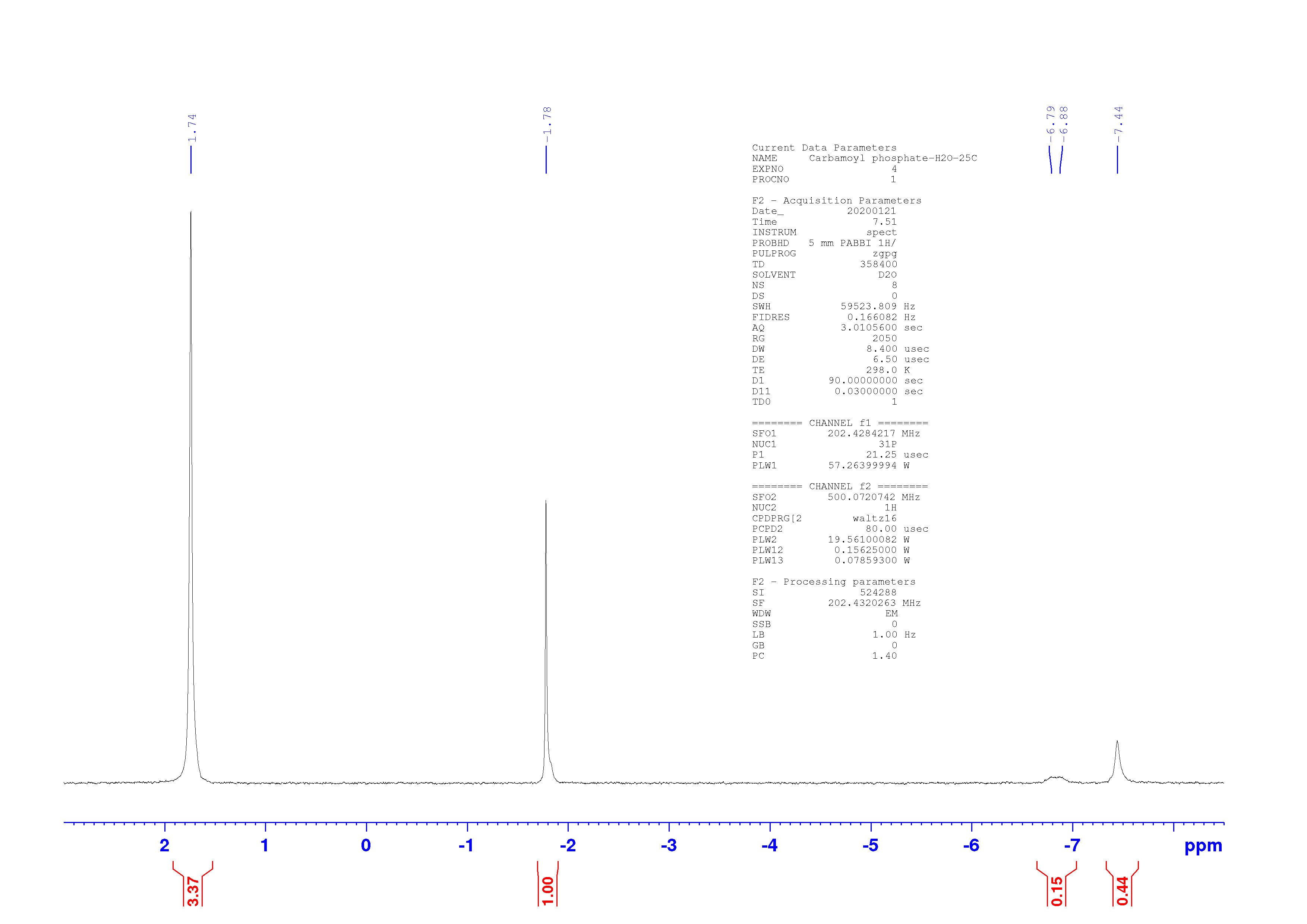


**Fig. S2** ^31^P NMR spectrum of carbamoyl phosphate in deuterated water at 25°C after 17h 10 min


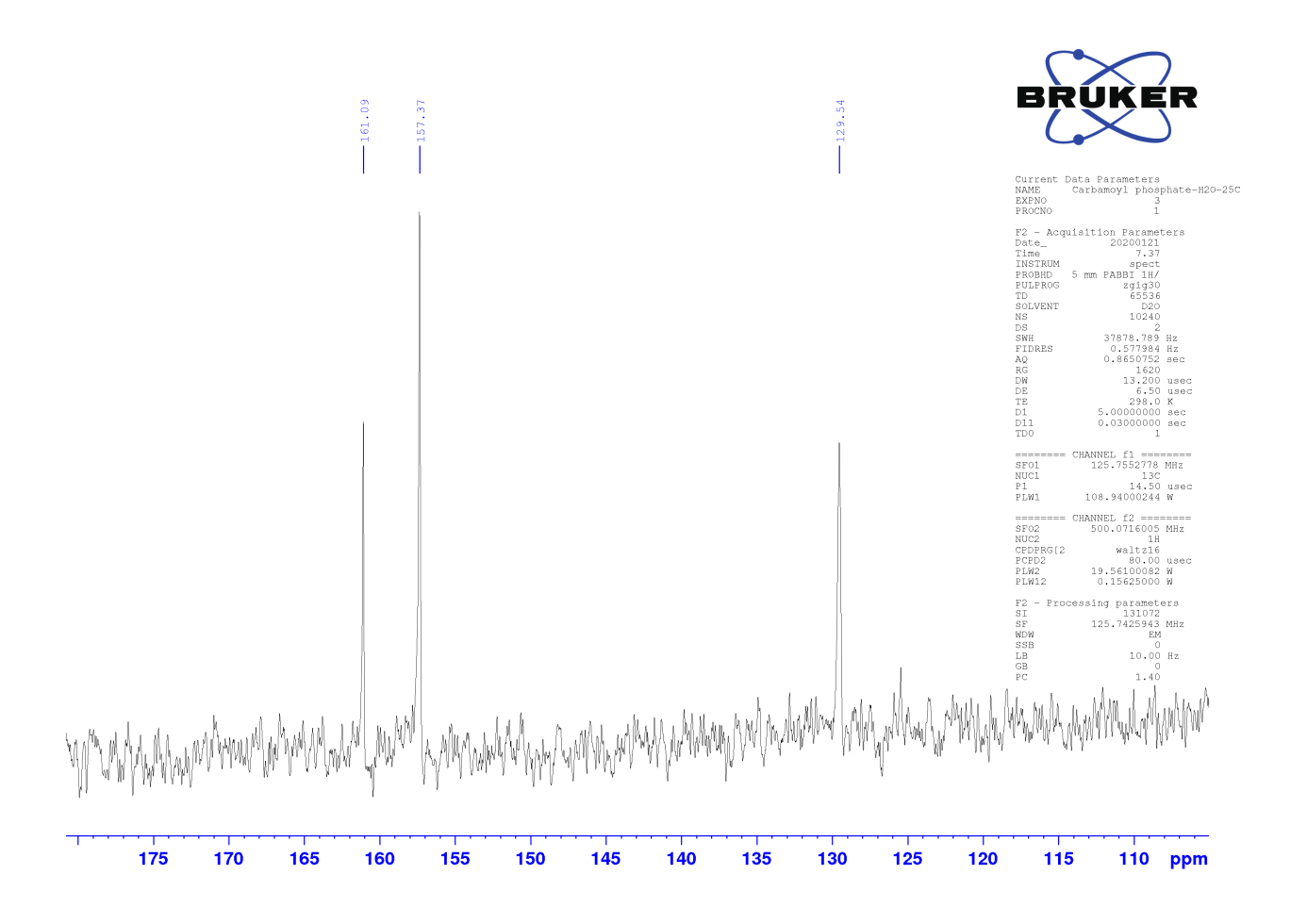


**Fig. S3** ^13^C NMR spectrum of carbamoyl phosphate in deuterated water at 25°C, accumulated between 1 and 17h.


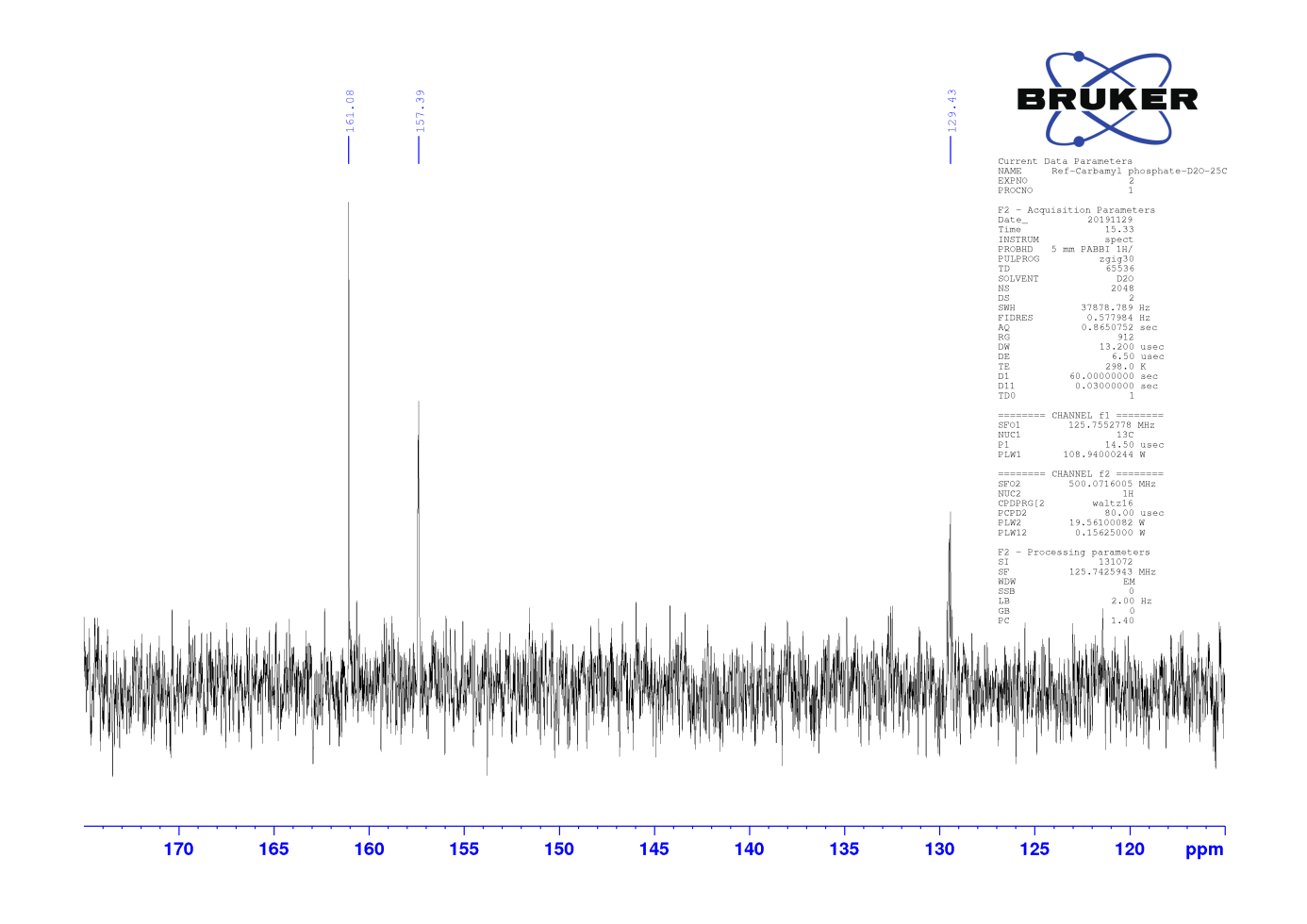


**Fig. S4** ^13^C NMR spectrum of carbamoyl phosphate in deuterated water at 25°C averaged between 18 and 35h


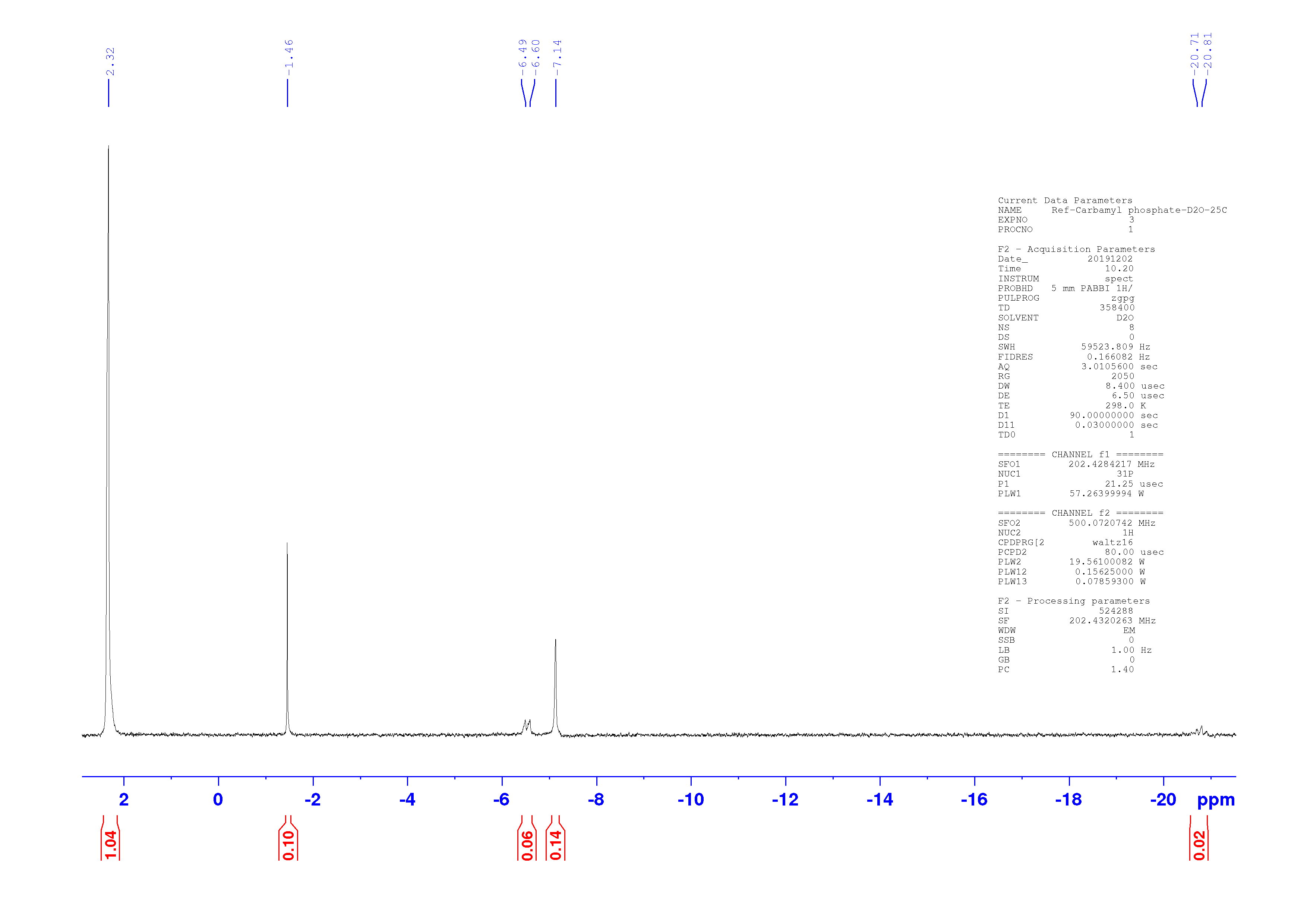


**Fig. S5** Final ^31^P NMR spectrum of carbamoyl phosphate in deuterated water after 3 days at 25°C

**Water + carbamoyl phosphate at 25°C**

ATR data:

After CP dissolution in water:

(Bruker, ATR, H_2_O, 25°C, cm^-1^) 2170 (cyanate, asymmetric stretch.), 2099 (O=P-OH stretch.), 1672 (CP, amide I, C=O ureide), 1601 (CP, amide II, NH_2_ bend. + O=P-OH stretch.), 1387 (CP, amide III, C-N stretch.), 1125 (CP, P=O stretch.), 1037 (CP, P-O stretch.), 974 (CP, P-O-C stretch. out-of-phase), 913 (P-O-C stretch. in-phase)

After 22h in water:

(Bruker, ATR, H_2_O, 25°C, cm^-1^) 2171 (cyanate, asymmetric stretch.), 1689 (CP, amide I, C=O ureide), 1587 (CP, amide II, NH_2_ bend. + O=P-OH stretch.), 1460 (?), 1381 (CP, amide III, C-N stretch.), 1122 (CP, P=O stretch.), 1082 (P_i_, P=O stretch.), 991 (MAP/ammonium phosphate, P-N stretch.), 977 (CP, P-O-C stretch. out-of-phase), 914 (P-O-C stretch. in-phase)

**Fig. S6** ATR-IR spectra of CP after dissolution (blue) and after 22h reaction (red)

**Kinetics:**

IR kinetics: t_1/2_ = 150 min for cyanate formation.

a)**
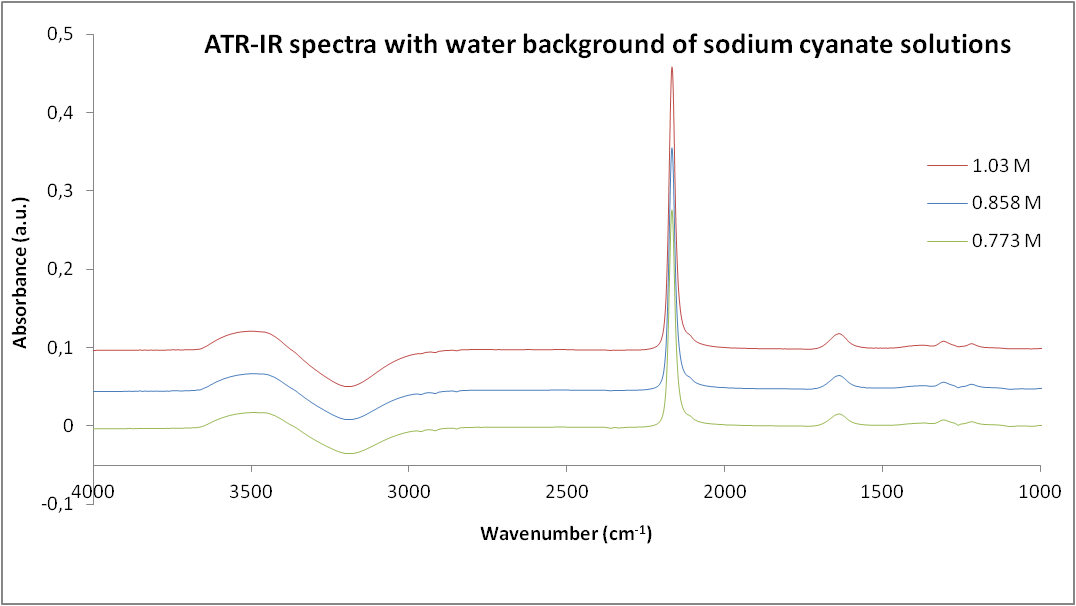
**

b)**
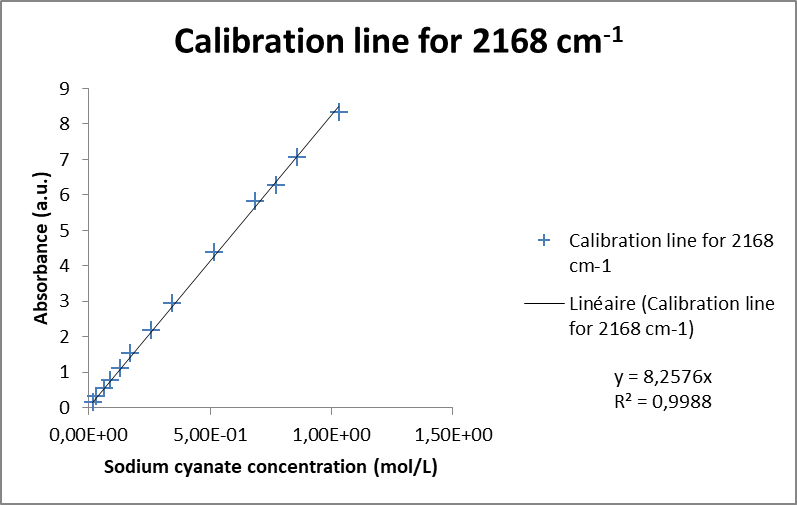
** c)
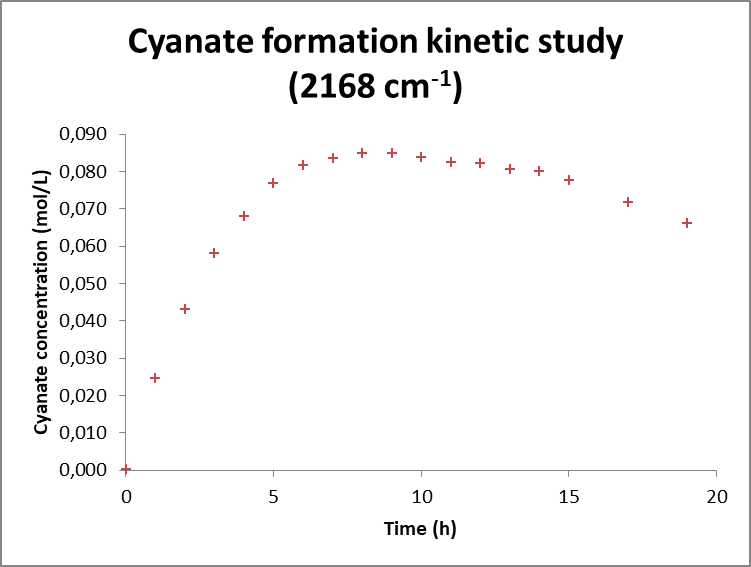


**Fig. S7** Kinetic evolution of cyanate formation followed and calibrated by ATR-IR

^31^P NMR kinetics:


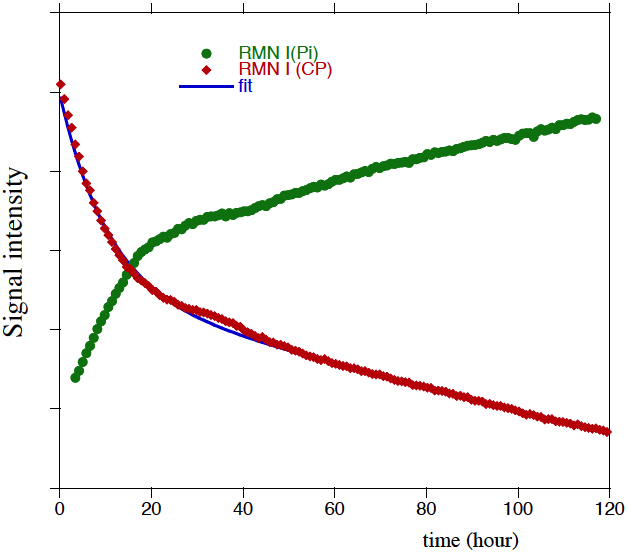


**Fig. S8** Quantitative kinetic evolution of carbamoyl phosphate (-1.55 ppm) and monophosphate (1.91 ppm) in deuterated water at 25°C

- 1. Carbamoyl phosphate ammonolysis

A**mmonia + carbamoyl phosphate at 25°C**

NMR data:

^31^P NMR (Bruker, 202.43 MHz, D_2_O, 25°C, ppm), t_0_ + 12 min acquisition

MAP: δ 8.45 (s); 1P: 3.48 (s); Carbamoyl phosphate: -1.24 (s); 3P: -4.90 (d); 2P: -5.36 (s); 3P: -19.35 (m)

^13^C NMR (Bruker, 125.74 MHz, D_2_O, 25°C, ppm), t_0_ + 23h 27 min

Carbonate: δ 168.11 (s); Carbamate: 166.63 (s); Urea: 163.76 (s); Cyanate: 129.58 (t, 10Hz)

^31^P NMR (Bruker, 202.43 MHz, D_2_O, 25°C, ppm), t_0_ + 23h 39 min

MAP: δ 8.44 (s); 1P: 3.46 (s); 3P: -4.94 (d, 20Hz); 2P: -5.40 (s); 3P: -19.38 (t)

pH: 11.26


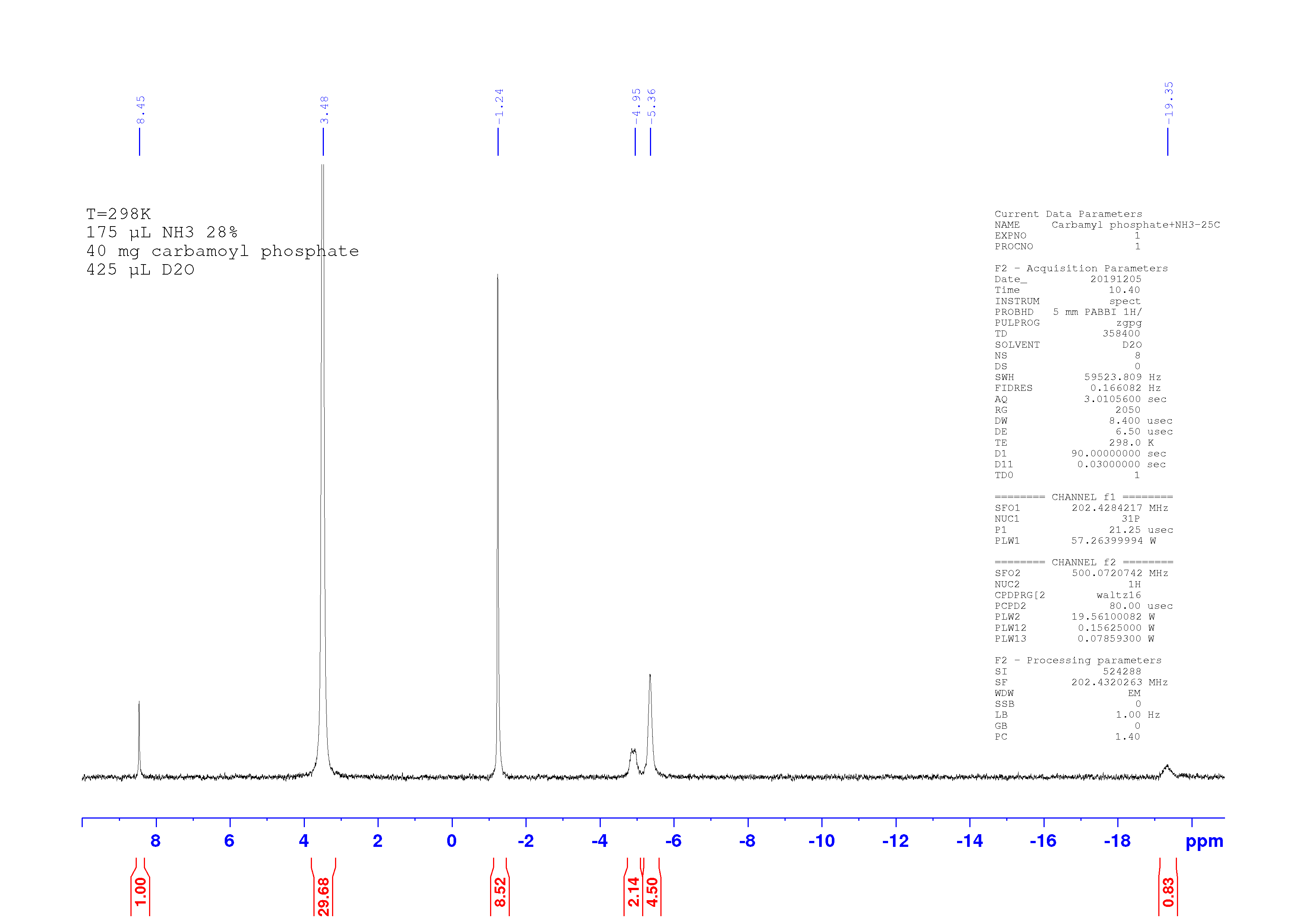


**Fig. S9** Initial ^31^P NMR spectrum of carbamoyl phosphate with ammonia in deuterated water at 25°C


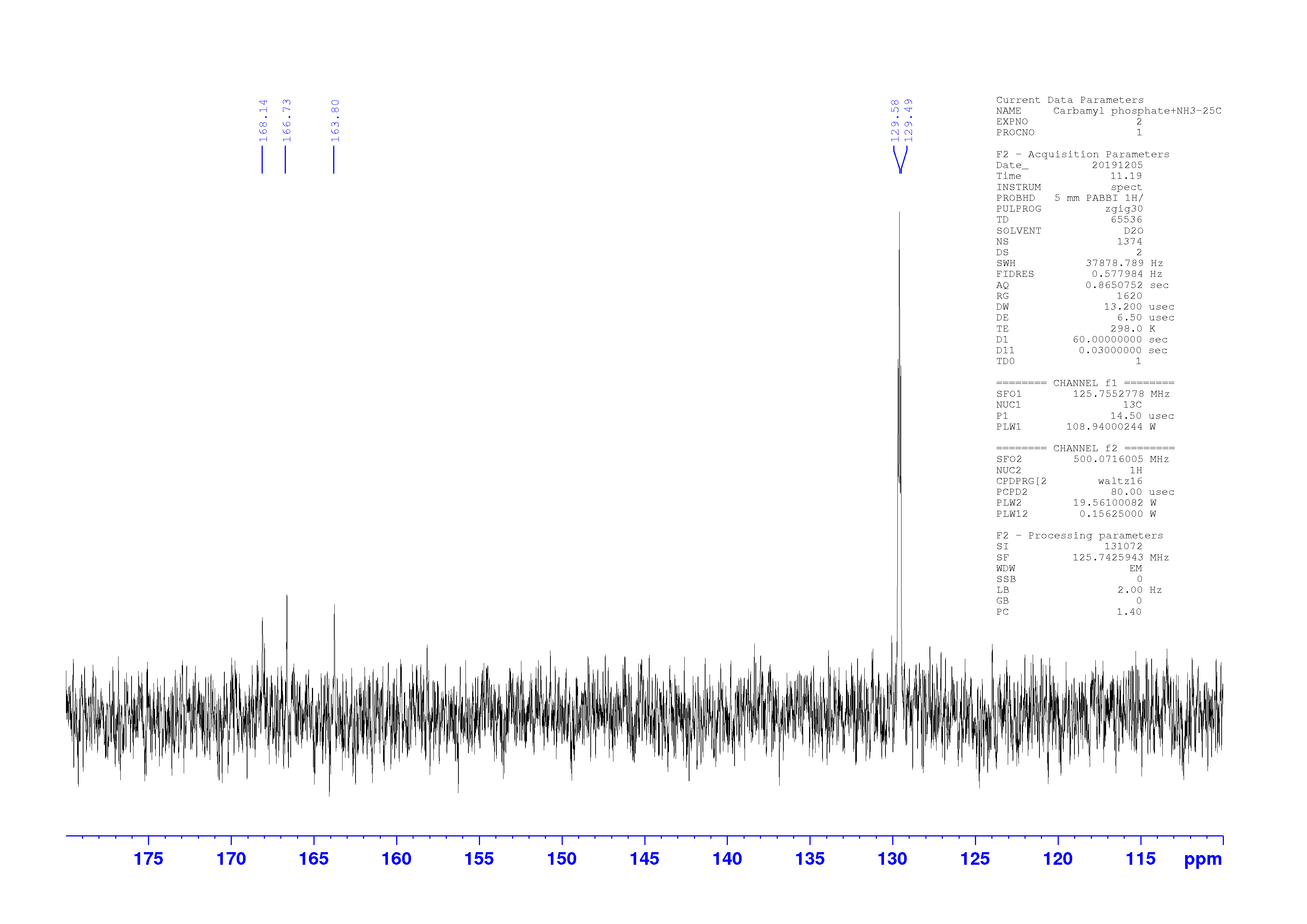


**Fig. S10** ^13^C NMR spectrum of carbamoyl phosphate with ammonia in deuterated water from 22 min to 23h 27 min at 25°C


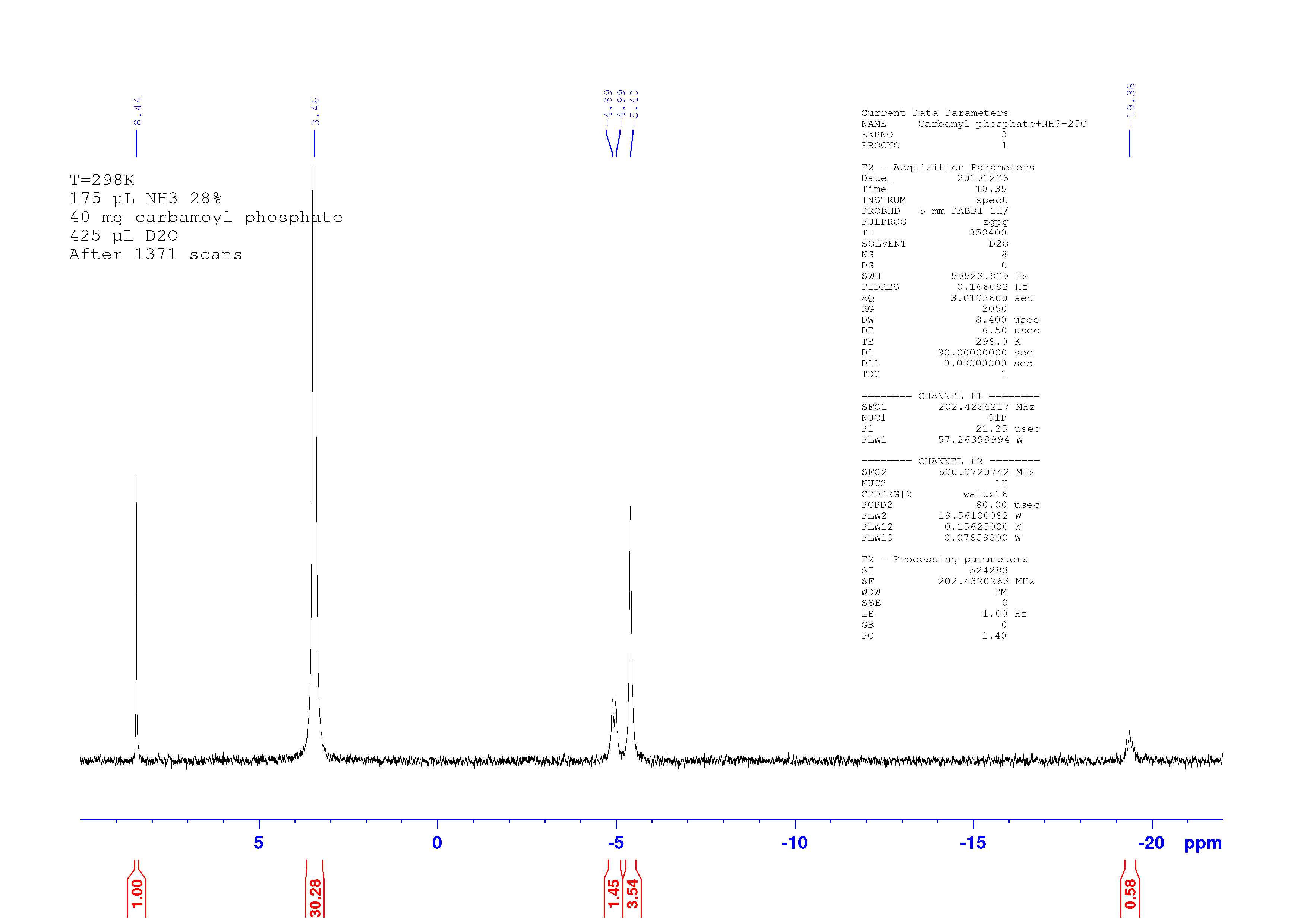


**Fig. S11** Final ^31^P NMR spectrum of carbamoyl phosphate with ammonia in deuterated water after 23h 39 min at 25°C

**Kinetics:**

IR kinetics: t_1/2_ = 7.25 min for cyanate formation.


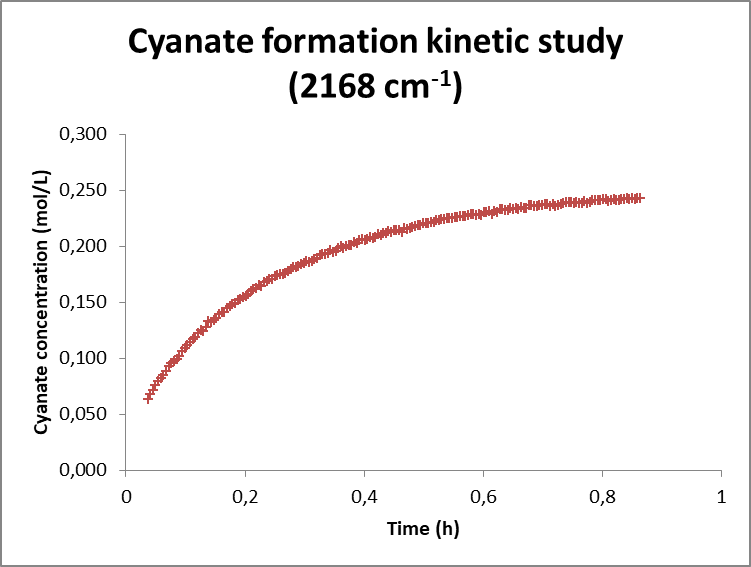


**Fig. S12** Kinetic evolution of cyanate in aqueous NH_3_, followed by ATR-IR

^31^P NMR kinetics:


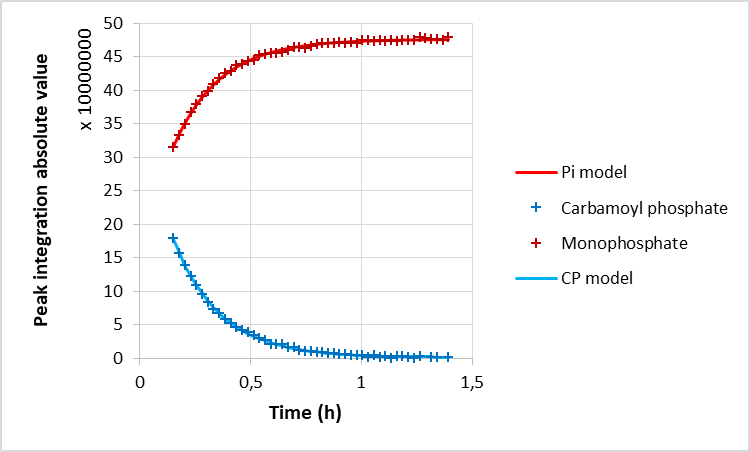


**Fig. S13** Quantitative kinetic evolution of carbamoyl phosphate (-1.29 ppm) and monophosphate (3.43 ppm) in aqueous ammonia at 25°C

**
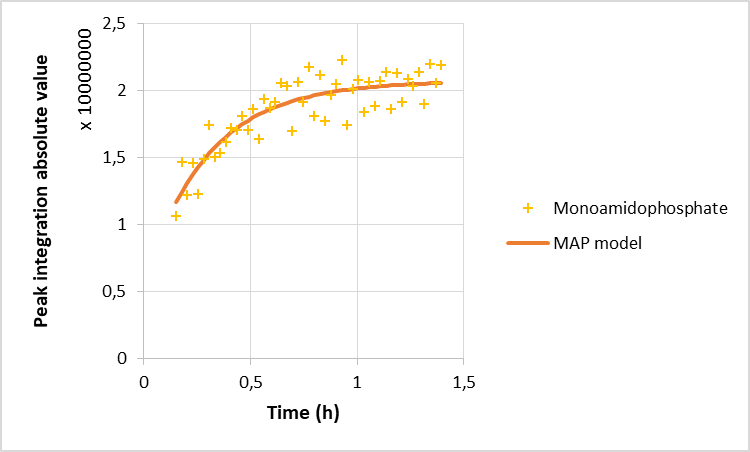
**

**Fig. S14** Quantitative kinetic evolution of monoamidophosphate (8.44 ppm) in aqueous ammonia at 25°C

Bruker report available on demand.

Model for CP: f(t) = Io * exp(-kt) + C with Io = 3.65E+8 ± 1.394E+6 , k = 0.0792 ± 2.278E-4 min^-1^ and C = 9.36E+5 ± 9.481E+4. t_1/2_ = 8.72 min for CP decomposition.

Model for P_i_: f(t) = Io * [1-exp(-kt)] + C with Io =3.32E+8 ± 1.426E+6, k = 0.0794 ± 3.310E-4 min^-1^ and C = 1.45E+8 ± 1.484E+6. t_1/2_ = 4.17 min for P_i_ formation.

Model for MAP: f(t) = Io * [1-exp(-kt)] + C with Io =1.49E+7 ± 9.077E+5, k = 0.0552 ± 4.756E-3 min^-1^ and C = 5.78E+6 ± 1.009E+6. t_1/2_ = 6.62 min for MAP formation.


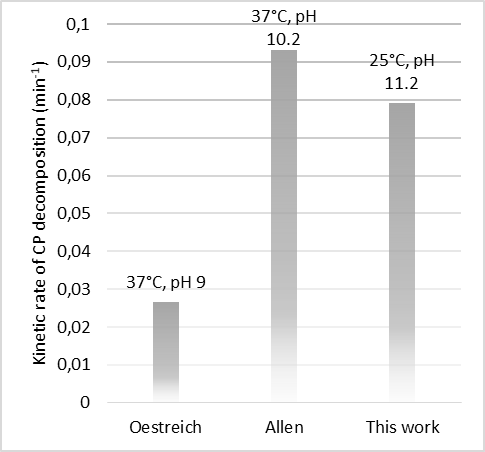


**Fig. S15** First order kinetic rates comparison for CP alkaline decomposition

^13^C NMR kinetics:


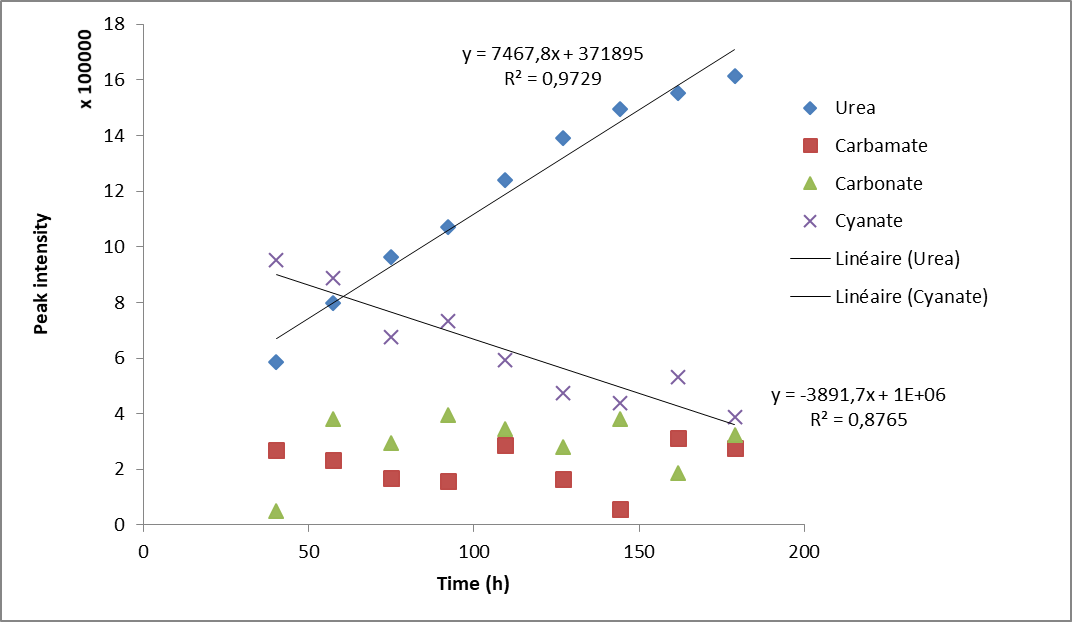


**Fig. S16** Qualitative kinetic evolution of cyanate (129 ppm), carbonate (168 ppm), carbamate (166 ppm) and urea (163 ppm) followed by ^13^C NMR

- 1. Trimetaphosphate stability in water or ammonia

**P3m in water after 45 days**

NMR data:

^31^P NMR (Bruker, 202.43 MHz, H_2_O, 30°C, ppm):

Initial: 2P: δ -6.98 (s ?); P3m: δ -21.10 (s) pH : 5.83

After 45 days: H_3_PO_4_: +0.29 (s); 3P: -8.26 (d, 19.7Hz), -9.07 (s); P3m: -21.19 (s)

**
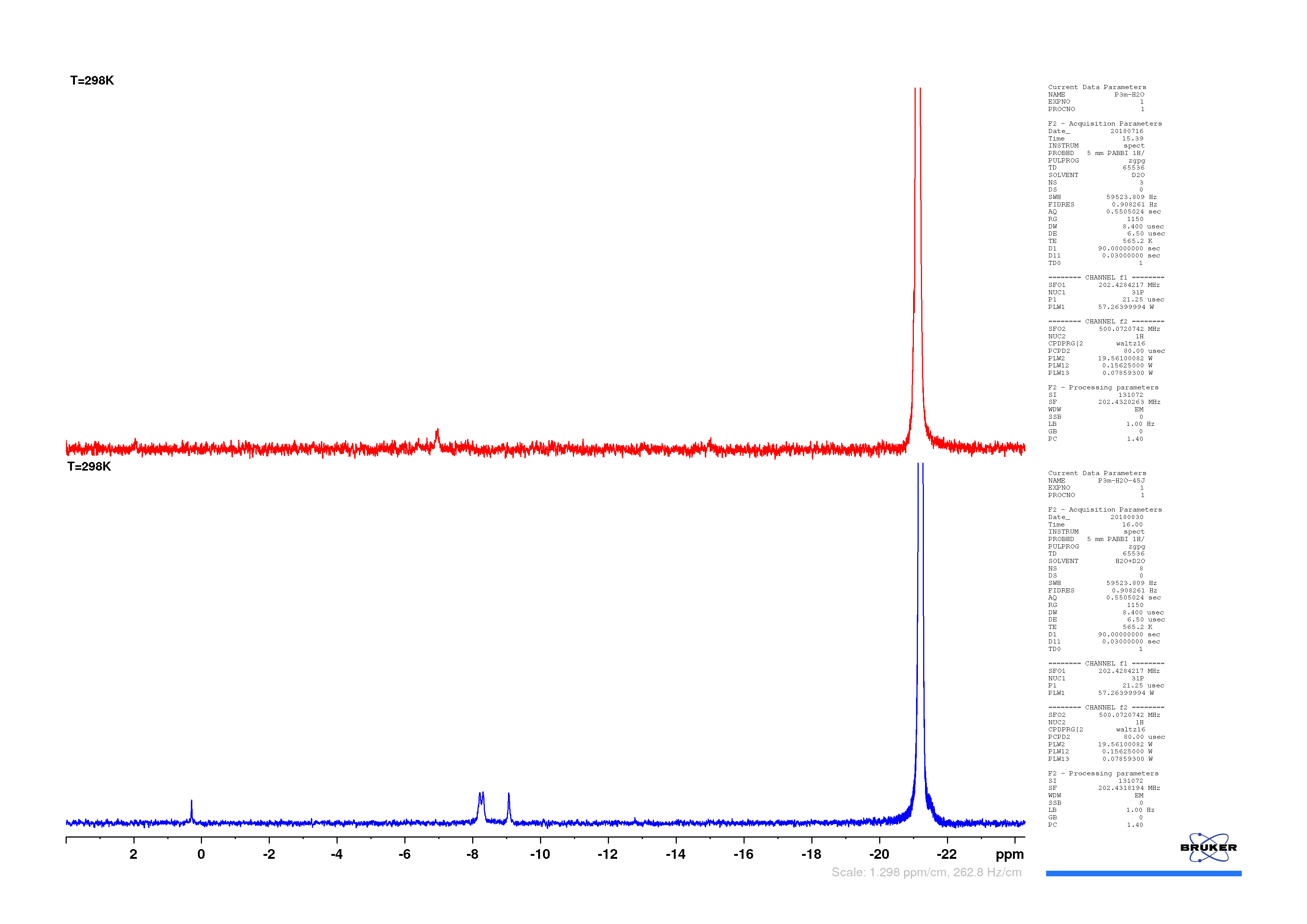
**

**Fig. S17** ^31^P NMR spectra of sodium trimetaphosphate in water after 0 (top) and 45 days (bottom) at 25°C

**Phosphoramidates formation by P3m opening, activation for 66h at 70°C**

NMR data:

^31^P NMR (Bruker, 202.43 MHz, H_2_O, 30°C, ppm):

DAP: δ 13.89 (s); MAP: δ 8.21 (s); 1P: δ 3.15 (s); MA3P: δ -0.24 (d, 19.2 Hz) -5.48 (d, 20.4 Hz), -20.74 (t, 19.8 Hz); MA2P: δ -0.64 (d, 19.2 Hz), -5.73 (d, 19.8 Hz); 2P: δ -5.81 (s). Unknown minority species: δ -5.33 (d, ~20 Hz), -19.75 (t, ~20 Hz)


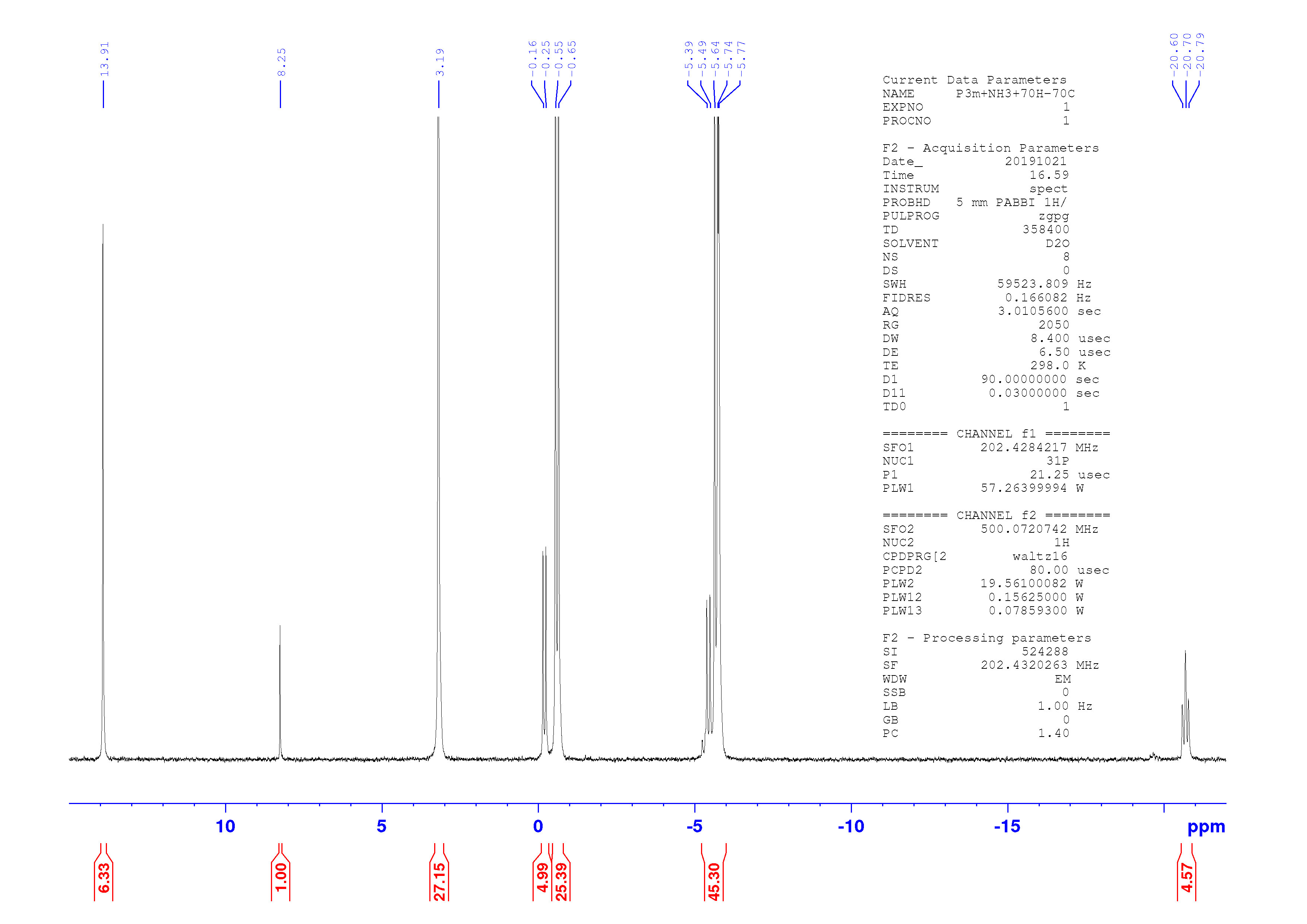


**Fig. S18** ^31^P NMR spectrum of ammonolysed sodium trimetaphosphate after 66h at 70°C

ATR data:

Phosphoramidate solution after 70h at 70°C:

(Bruker, ATR, H_2_O, 25°C, cm^-1^) 1460 (ammonium, –NH bend.), 1192 (phosphoramidates, P=O stretch.), 1095/1082/1013 (phosphoramidates, P-N/P-O stretch.), 990 (phosphoramidates, P-N stretch.), 932 (phosphoramidates, P-N stretch.), 854 (?)


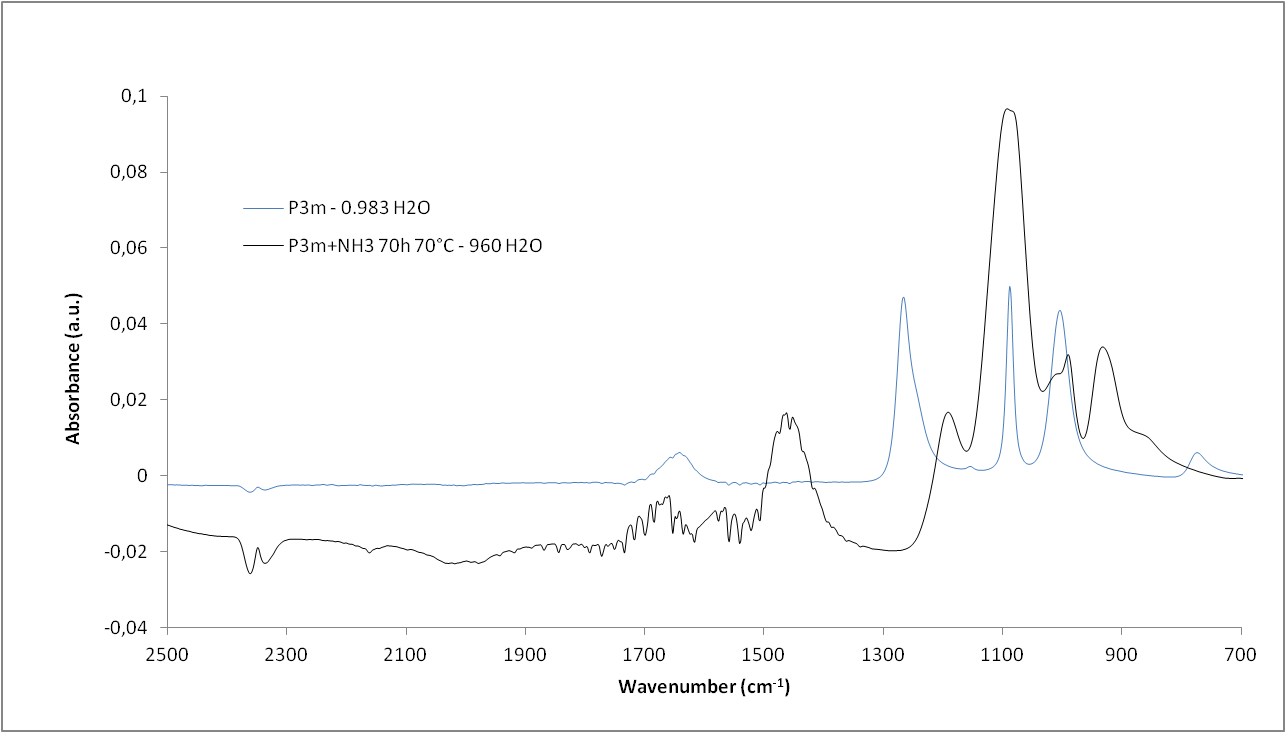


**Fig. S19** ATR-IR spectra of P3m in aqueous solution (spectrum of water subtracted as background) and of ammonolysed P3m (70h at 70°C, aqueous ammonia subtracted as background).

**Kinetics:**

^31^P NMR kinetics:


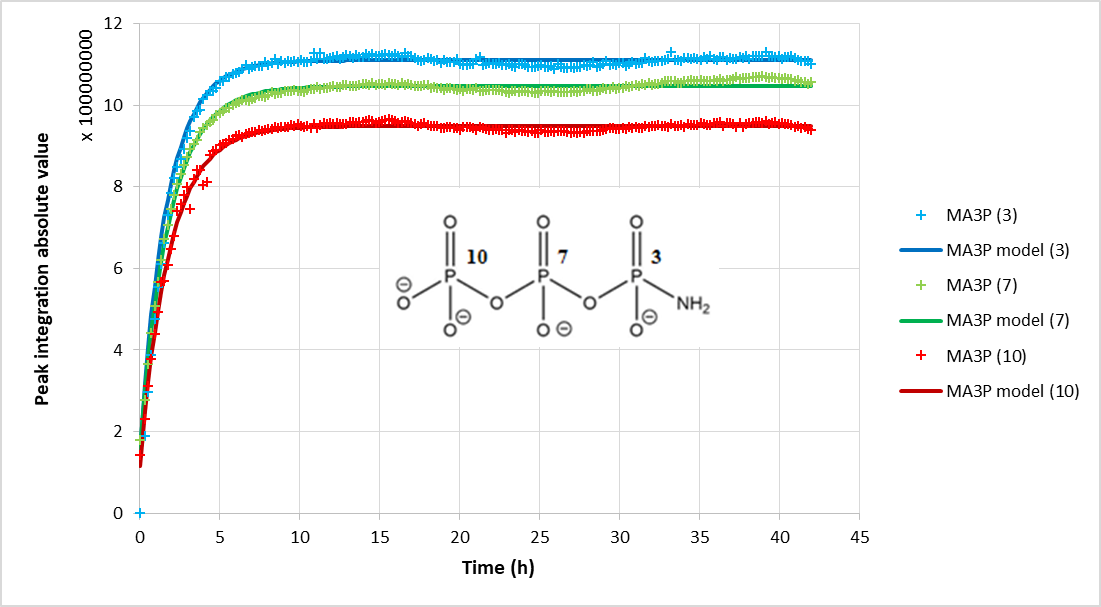


**Fig. S20** Evolution of the three ^31^P NMR signals assigned to MA3P (-0.09, -5.07 and -20.26 ppm) in aqueous ammonia at 30°C.


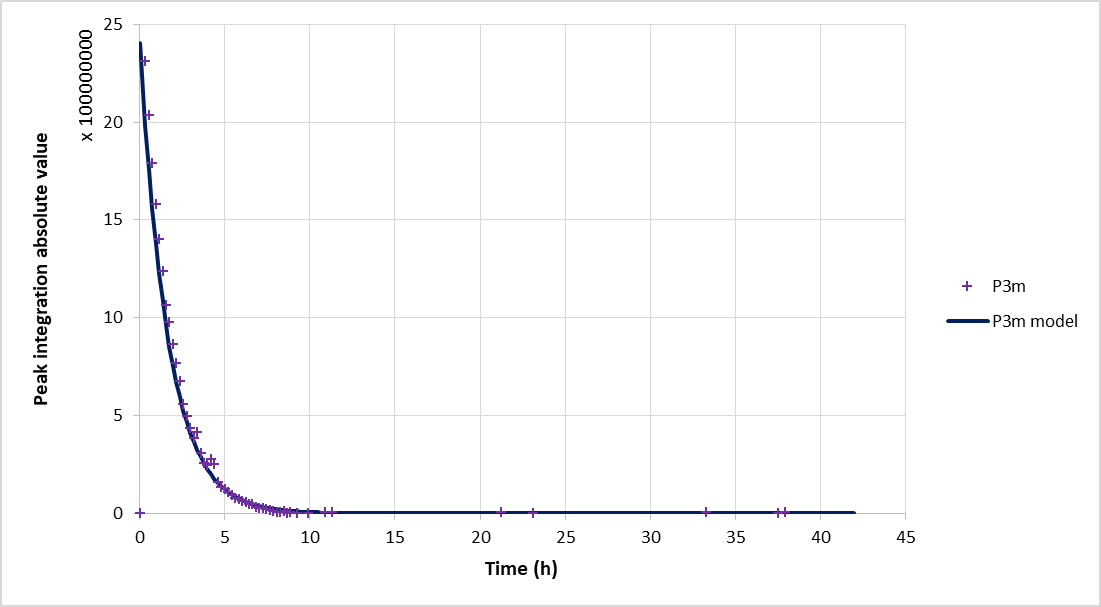


**Fig. S21** Evolution of the P3m signal (-21.48 ppm) in aqueous ammonia at 30°C

Bruker report available on demand.

Model for MA3P (3): f(t) = Io * [1-exp(-kt)] + C with Io = 9.62E+8 ± 7.537E+5 , k = 0.590 ± 6.852E-4 h^-1^ and C = 1.49E+8 ± 7.576E+5. t_1/2_ = 55 min for MA3P (3) formation.

Model for MA3P (7): f(t) = Io * [1-exp(-kt)] + C with Io = 8.88E+8 ± 9.270E+5 , k = 0.537 ± 8.330E-4 h^-1^ and C = 1.60E+8 ± 9.325E+5. t_1/2_ = 59 min for MA3P (7) formation.

Model for MA3P (10): f(t) = Io * [1-exp(-kt)] + C with Io = 8.35E+8 ± 9.000E+5 , k = 0535 ± 8.579E-4 h^-1^ and C = 1.14E+8 ± 9.053E+5. t_1/2_ = 63 min for MA3P (10) formation.

Model for P3m: f(t) = Io * exp(-kt) with Io = 2.41E+9 ± 8.101E+5 and k = 0.593 ± 2.822E-4 h^-1^. t_1/2_ = 74 min for P3m opening.

- 1. Trimetaphosphate evolution in presence of carbonylated compounds

**P3m + ammonium carbamate after 3h at 25°C**

NMR data:

^31^P NMR (Bruker, 202.43 MHz, D_2_O, 25°C, ppm), 12 min acquisition after reacting for 3h.

1P: δ 2.40; MA3P: -0.41 (d, 19Hz); Carbamoyltriphosphate: -0.46 (d, 19Hz); MA3P + Carbamoyltriphosphate: -6.18 (d, 20Hz); 2P: -6.60 (s); MA3P: -21.13 (t, 20Hz); Carbamoyltriphosphate: -21.33 (masked t); P3m: -21.43 (s)


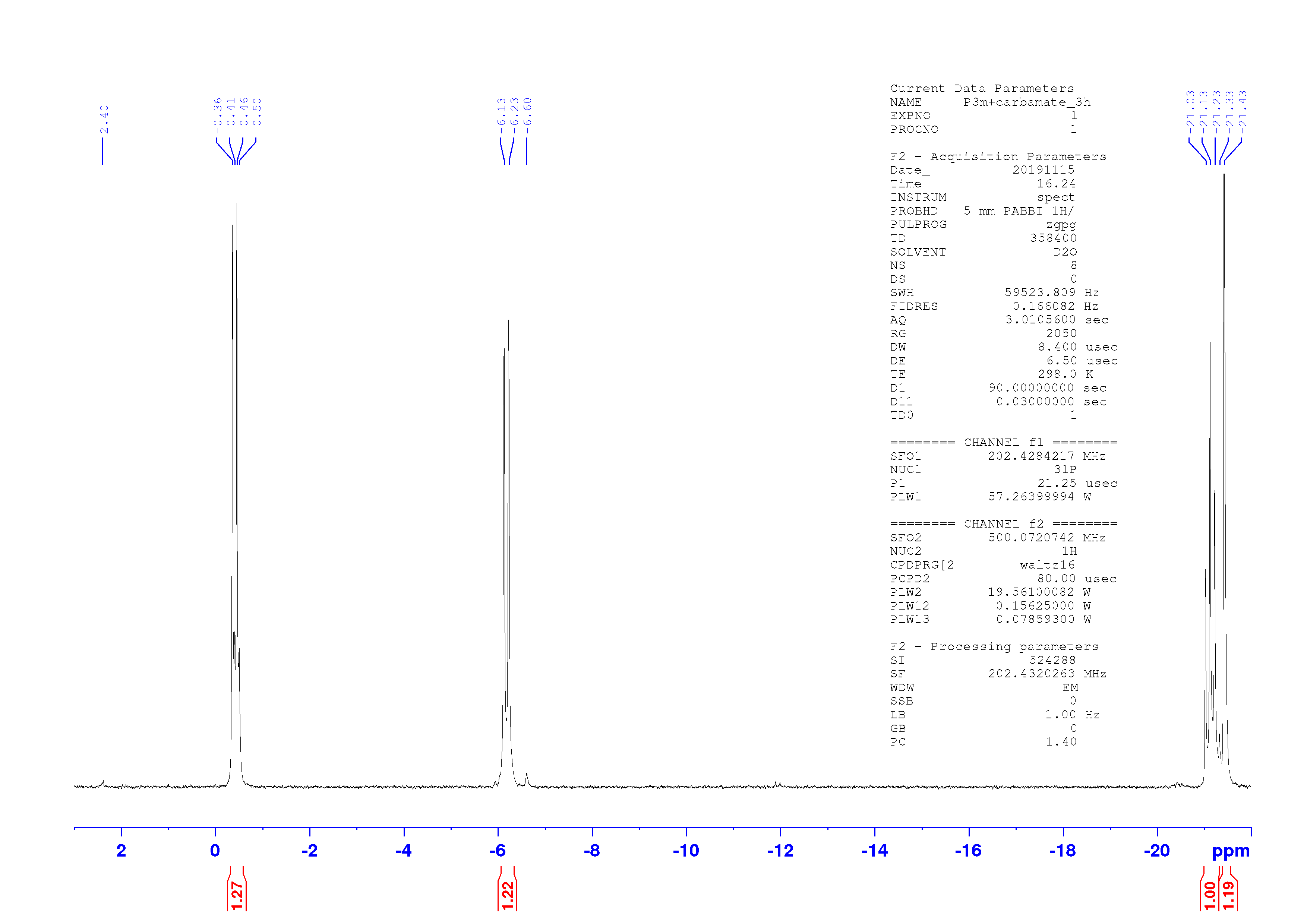


**Fig. S22** ^31^P NMR spectrum of sodium trimetaphosphate with ammonium carbamate in deuterated water after 192 minutes at 25°C

**P3m + ammonium carbamate after 1 week at 25°C**

NMR data:

^31^P NMR (Bruker, 202.43 MHz, D_2_O, 25°C, ppm), 12 min acquisition after ageing for one week

DAP: δ 13.72 (s); MAP: 7.19 (s); 1P: 2.27; MA3P: -0.53 (d, 19Hz); unknown: -6.02 (s); MA3P: -6.34 (d, 20Hz); 2P: -6.79 (s); unknown: -12.11 (d, 19Hz); -20.67 (t, 20Hz); MA3P: -21.32 (t, 20Hz); P3m: -21.56 (s)

^13^C NMR (Bruker, 125.74 MHz, D_2_O, 25°C, ppm), acquisition for 3h 43 min after ageing for one week (recycle time = 3s, not quantitative)

Carbamate: δ 165.34 (s); Urea: 163.13 (s)

pH measure: 9.48


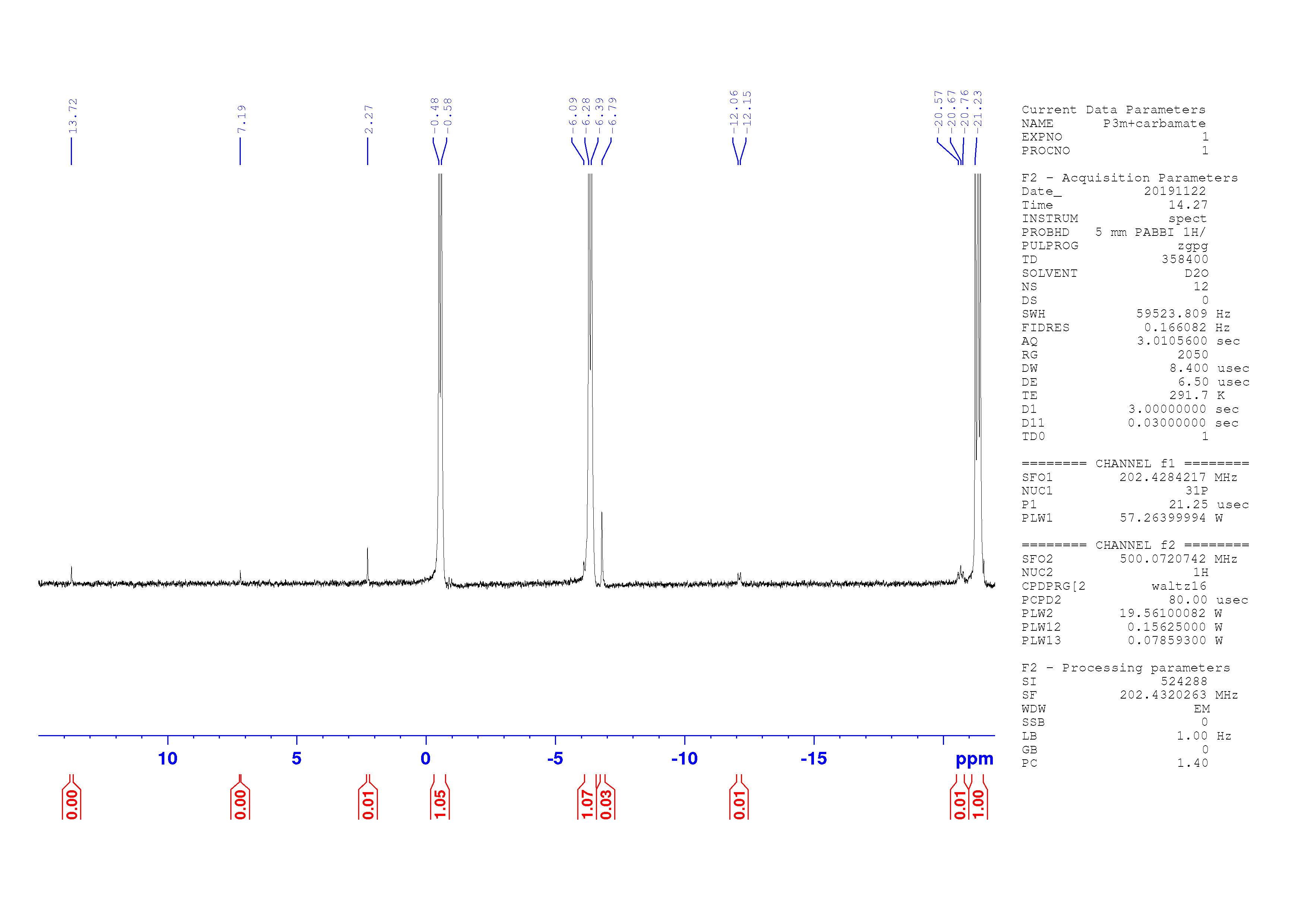


**Fig. S23** ^31^P NMR spectrum of sodium trimetaphosphate with ammonium carbamate in deuterated water after 1 week at 25°C

**
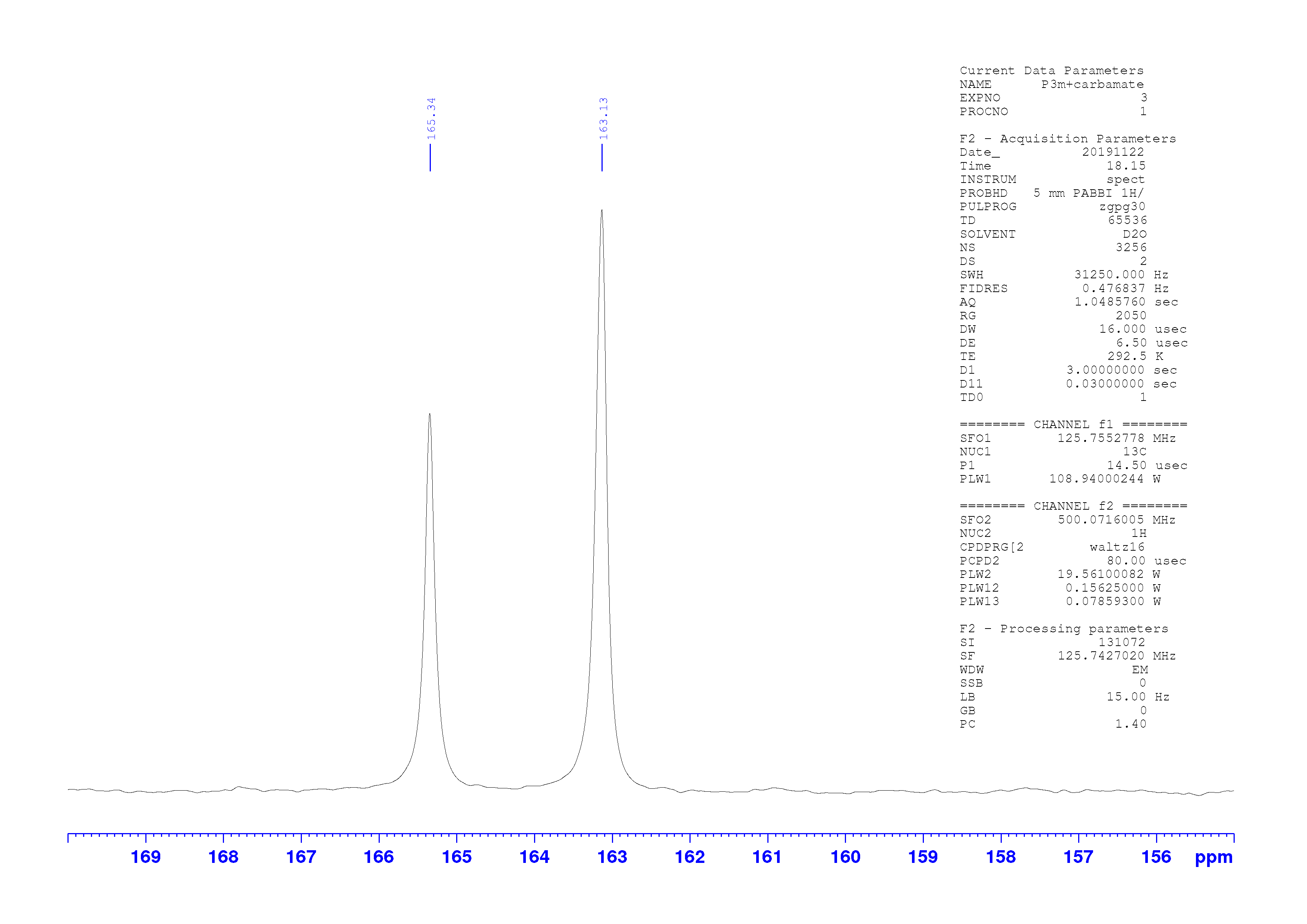
**

**Fig. S24** ^13^C NMR spectrum of sodium trimetaphosphate with ammonium carbamate in deuterated water after 1 week at 25°C

**P3m + urea after 70h at 70°C**

NMR data:

^31^P NMR (Bruker, 202.43 MHz, D_2_O, 25°C, ppm), 12 min acquisition after reacting 70h at 70°C

1P: δ 2.55 (s); 3P: -5.91 (d, 20Hz); 2P: -6.64 (s); 3P: -20.09 (t, 20Hz); P3m: -21.15 (s)

pH measure before heating: 6.5. pH measure after heating: 7.89


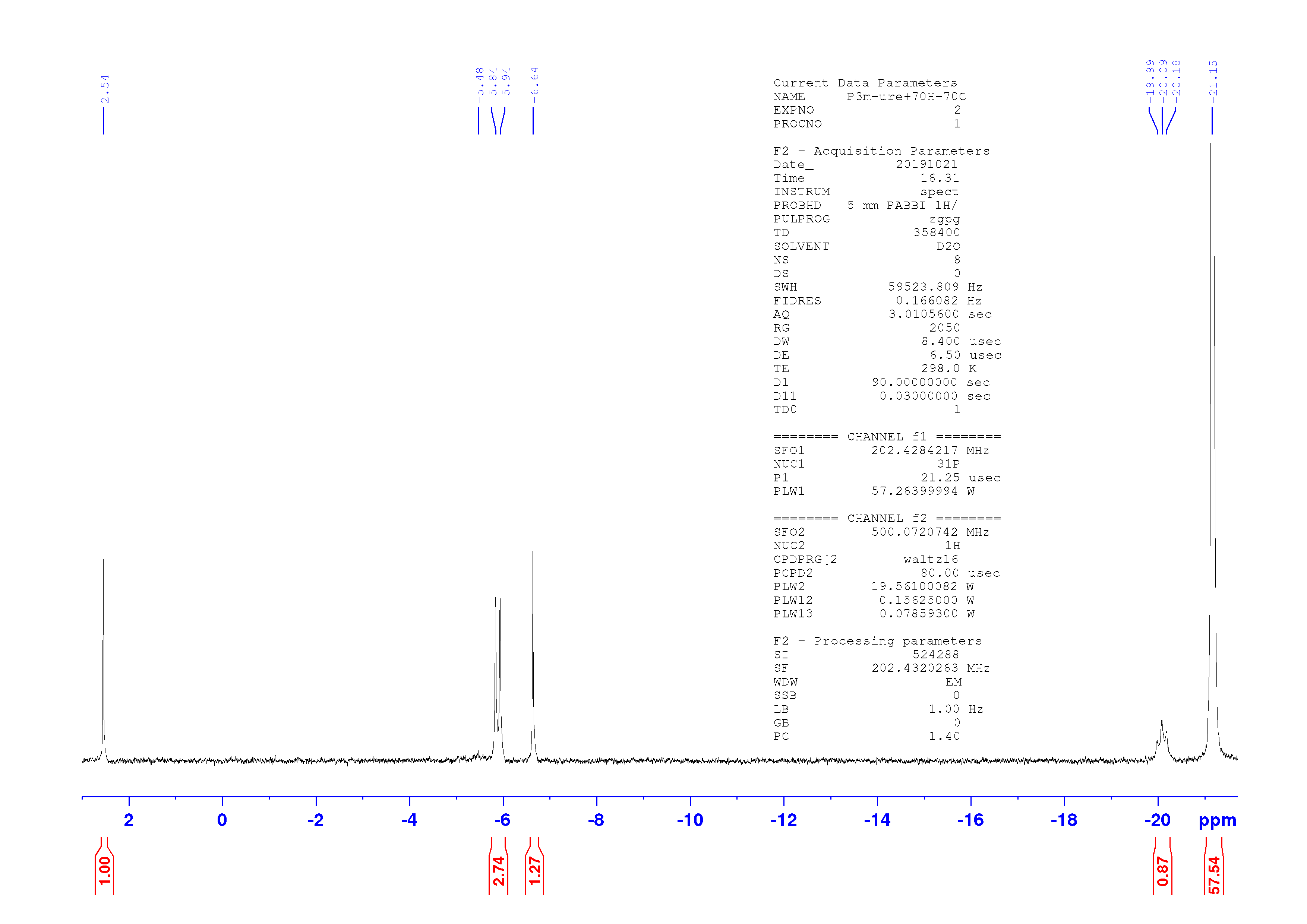


**Fig. S25** ^31^P NMR spectrum of sodium trimetaphosphate with urea in deuterated water after 70h at 70°C

**P3m + urea after 70h at 70°C + ammonium carbamate for 1.5 day at 25°C**

NMR data:

^31^P NMR (Bruker, 202.43 MHz, D_2_O, 25°C, ppm), 12 min acquisition after reacting 1.5 day at 25°C

1P: δ 2.61 (s); MA3P: - 0.32 (d, 19Hz); 3P: -5.72 (d, 19Hz); MA3P: - 5.79 (d, 20Hz); 2P: -6.25 (s); 3P: -20.16 (t, 19Hz); MA3P: -20.97 (t, 20Hz); P3m: -21.26 (s)


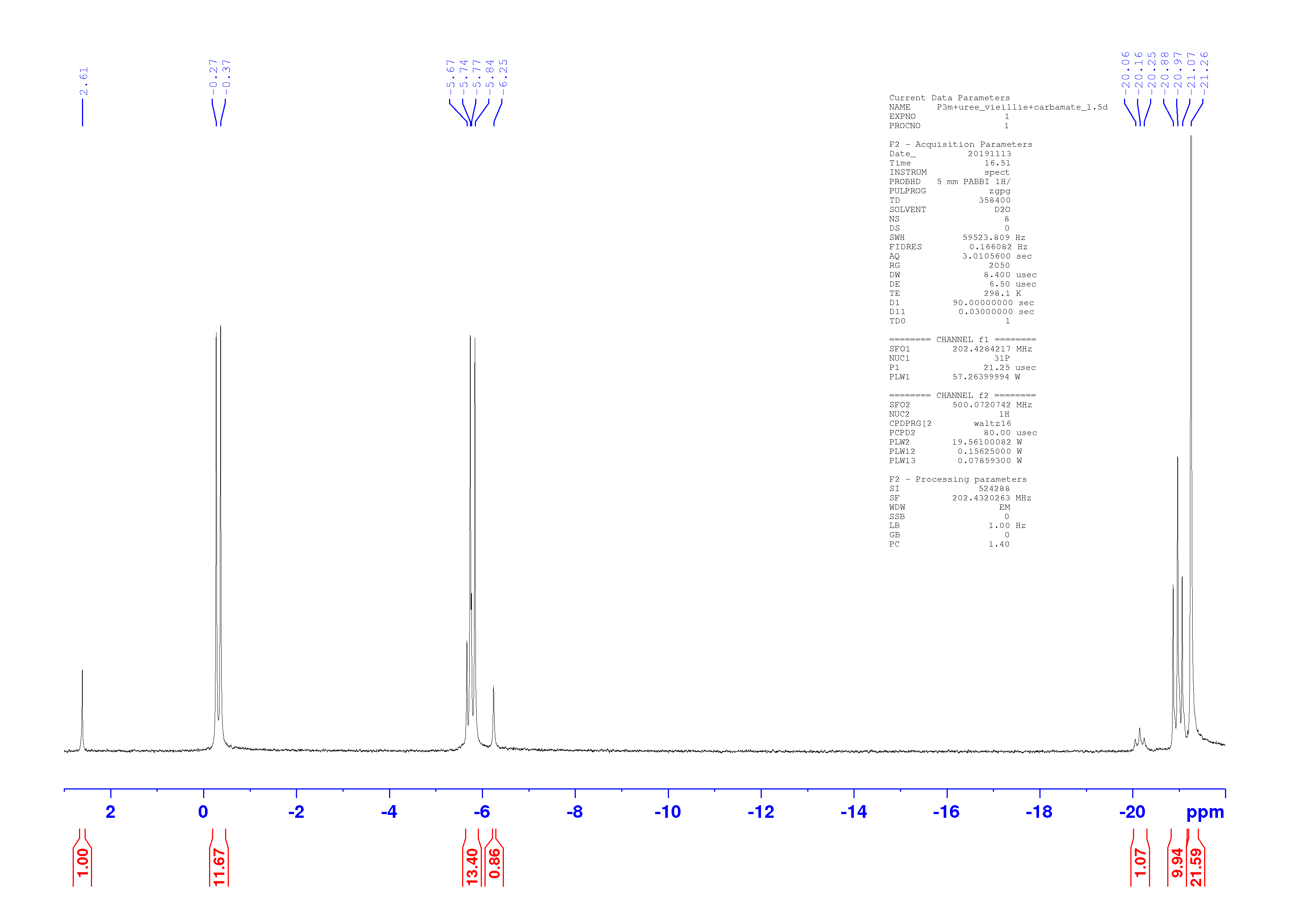


**Fig. S26** ^31^P NMR spectrum of sodium trimetaphosphate with urea in deuterated water after 70h at 70°C, then contacted with ammonium carbamate and aged for 1.5 d at 25°C.

**P3m + urea after 70h at 70°C + ammonium carbamate for 3.5 days at 25°C**

NMR data:

^31^P NMR (Bruker, 202.43 MHz, D_2_O, 25°C, ppm), 12 min acquisition after reacting 3.5 day at 25°C

1P: δ 2.61 (s); MA3P: - 0.35 (d, 19Hz); 3P: -5.74 (d, 20Hz); MA3P: - 5.80 (d, 20Hz); 2P: -6.28 (s); 3P: -20.21 (t, 20Hz); MA3P + carboxytriphosphate (?): -21.01 (t + masked t, 20Hz); P3m: -21.27 (s)

pH measure: 9.02


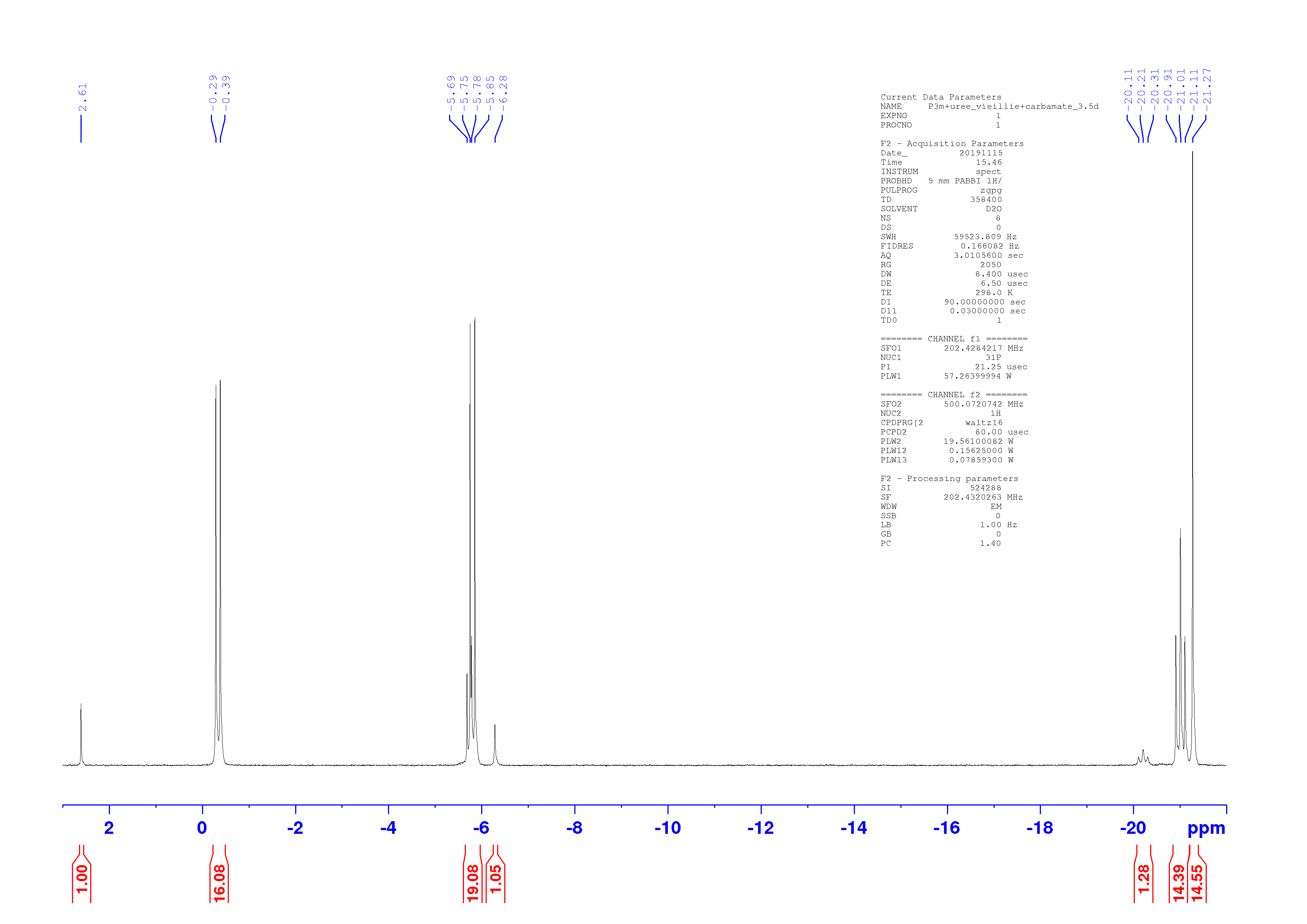


**Fig. S27** ^31^P NMR spectrum of sodium trimetaphosphate with urea in deuterated water after 70h at 70°C, then aged for 3.5 days after adding ammonium carbamate

ATR data:

Sodium trimetaphosphate with urea in water after 70h at 70°C, then aged for 3.5 days after ammonium carbamate addition:

(ATR, H_2_O, 25°C, cm^-1^) 1603 cm^-1^ (carbamate, C=O stretch.), 1537 cm^-1^ (carbamate, –N-H bend.), 1449 (ammonium, –NH bend.), 1360 (carbamate/carbonate stretch.), 1212 cm^-1^ (MA3P/carboxyphosphate, P=O stretch.), 1107 (ammonia, NH_3_ bend.), 1077 (phosphate, P-O stretch.), 1041 (carboxyphosphate, P-O-C stretch.), 1018 (MA3P, P-N stretch.), 990 (MA3P, P-N stretch.), 922 (phosphate-type, P-O-P as. stretch.), 878 (phosphate-type), 830 (carbamate/carbonate)


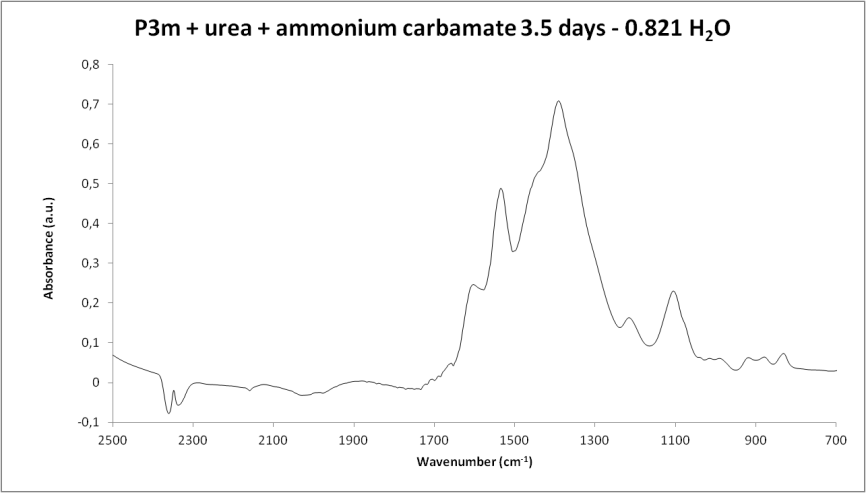


**Fig S28.** ATR-IR spectrum of sodium trimetaphosphate with urea in water after 70h at 70°C then aged for 3.5 days at 25°C after ammonium carbamate addition. The contribution of water was removed.


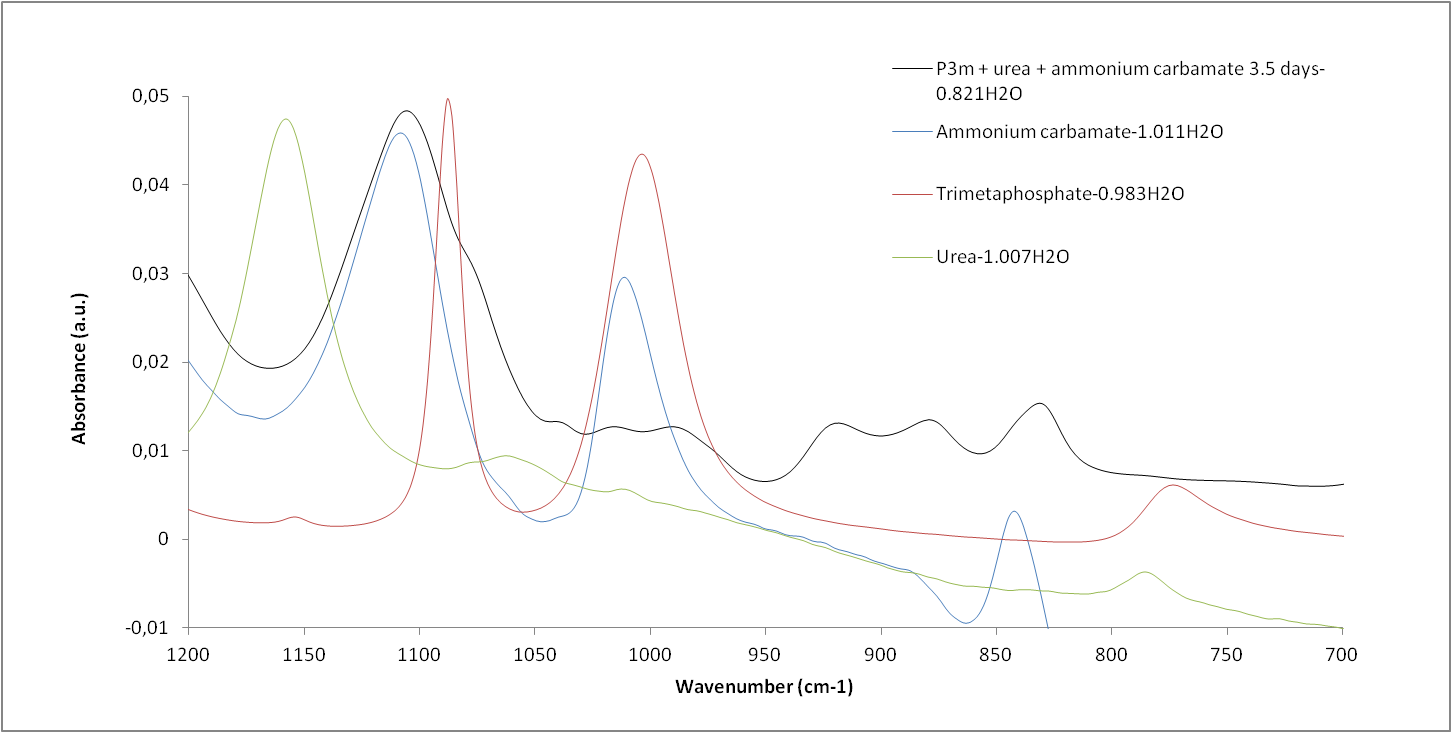


**Fig S29.** ATR-IR spectra comparison with normalisation of the references to the same intensity in the 1200-900 cm^-1^ rage (experiment x0.21, ammonium carbamate x10, P3m x1 and urea x7).

**P3m + ammonium carbonate after 2h at 25°C**

NMR data:

^31^P NMR (Bruker, 202.43 MHz, D_2_O, 25°C, ppm), 12 min acquisition after reacting 2h at 25°C

1P: δ 2.38 (s); MA3P: - 0.39 (d, 19Hz); -6.18 (d, 19Hz); 2P: -6.66 (s); and -21.29 (s) (?): ; P3m: -21.42 (s)

pH : 8.18


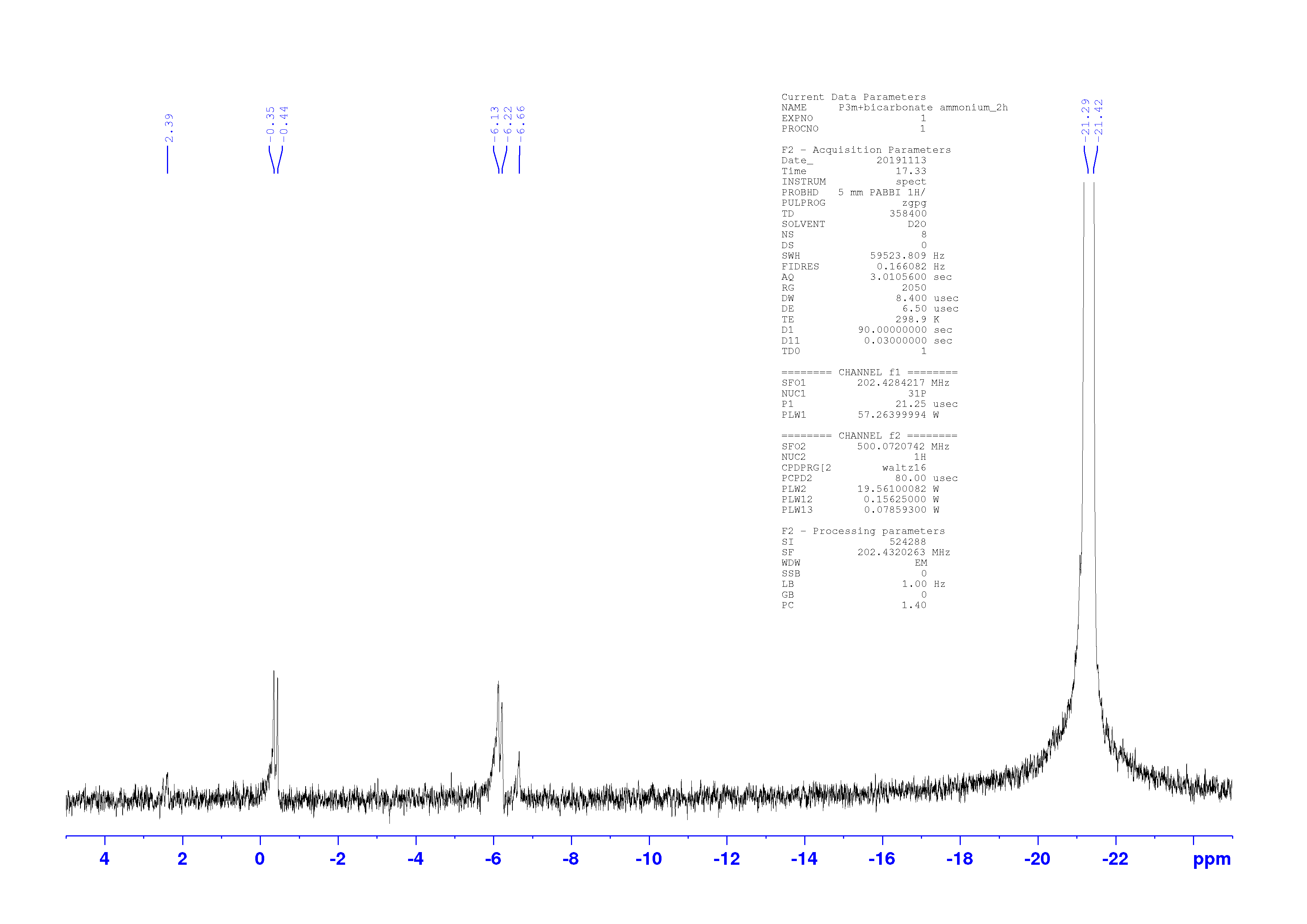


**Fig. S30** ^31^P NMR spectrum of sodium trimetaphosphate with ammonium carbonate in deuterated water after 2h at 25°C.

**P3m + ammonium carbonate after 2 days at 25°C**

NMR data:

^31^P NMR (Bruker, 202.43 MHz, D_2_O, 25°C, ppm), 12 min acquisition after 2 days reacting at 25°C

1P: δ 2.35 (s); MA3P: -0.43 (d, 19Hz); MA3P: -6.21 (d, 20Hz); 2P: -6.77 (s); MA3P: -21.23 (t, 20Hz); P3m: -21.45 (s)

pH, measured 8.05; inferred from monophosphate peak position, 8.00


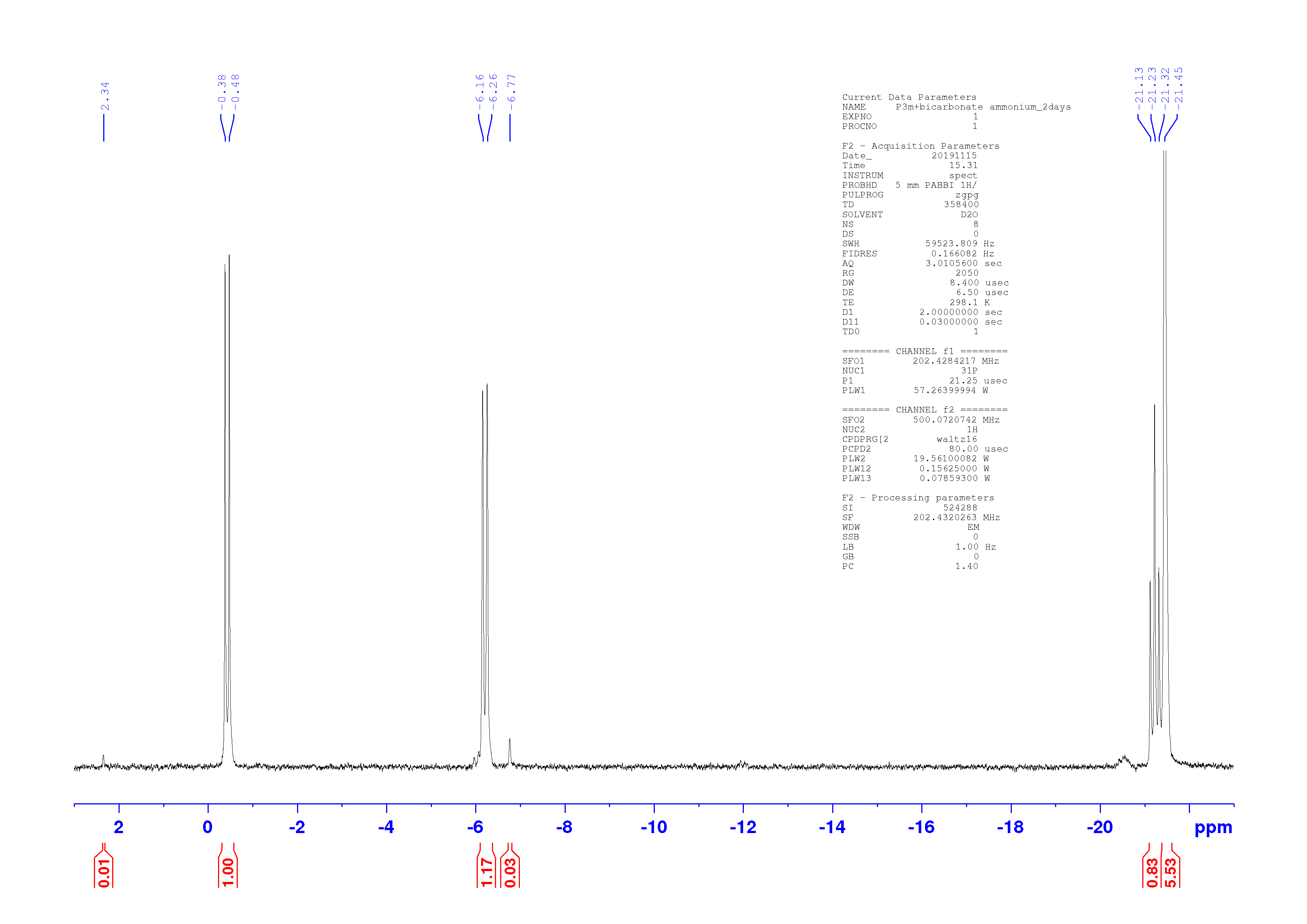


**Fig. S31** ^31^P NMR spectrum of sodium trimetaphosphate with ammonium carbonate in deuterated water after 2 days at 25°C

ATR data:

Sodium trimetaphosphate with ammonium carbonate in water after 2 days at 25°C:

(Bruker, ATR, H_2_O, 25°C, cm^-1^) 2344 (dissolved CO_2_, O=C=O stretching), 1613 (carbonate, C=O sym. stretch.), 1449 (ammonium, –NH bend.), 1358 (carbonate, C=O antisym. stretch. ), 1267 (P3m, O-P-O as. stretch.), 1223 (shoulder), 1111 (ammonia, NH_3_ bend.), 1088 (P3m, O-P-O sym. stretch.), 1000 (P3m, P-O-P antisym. stretch.), 924 (phosphoramidates, P-N sym. stretch.), 841 (carbonate, C-O sym. in-plane bending), 774 (P3m, P-O-P sym. stretch.)


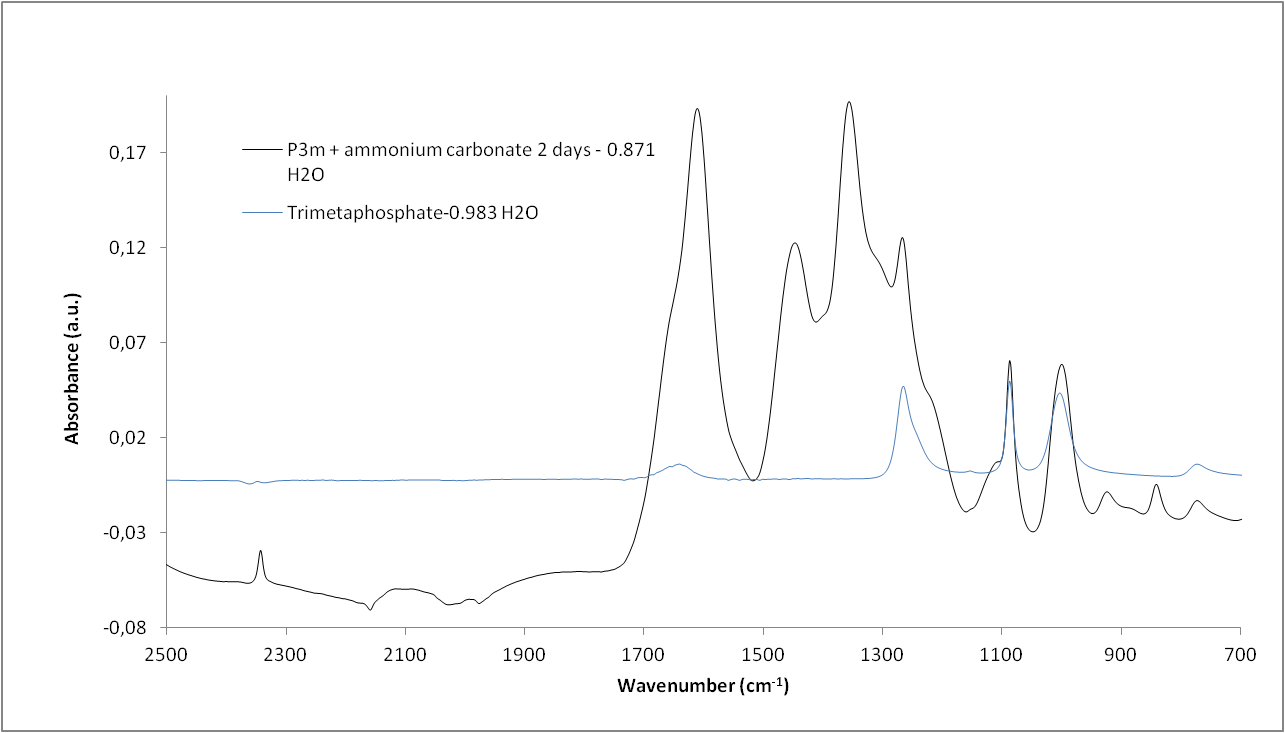


**Fig S32.** ATR-IR spectrum of sodium trimetaphosphate with ammonium carbonate in water after 2 days at 25°C. Water spectrum was removed.

**P3m + ammonium carbonate after 4 days at 25°C**

NMR data:

^31^P NMR (Bruker, 202.43 MHz, D_2_O, 25°C, ppm), 12 min acquisition after 4 days ageing at 25°C

1P: δ 2.26 (s); MA3P: -0.48 (d, 19Hz); MA3P: -6.29 (d, 20Hz); 2P: -6.83 (s); MA3P: -21.30 (t, 20Hz); P3m: -21.52 (s)

Unassigned: -6.07 (s); -6.29 (d, 19.7Hz); -12.05 (d, around 18Hz), -20.63 (t, 19.5Hz)


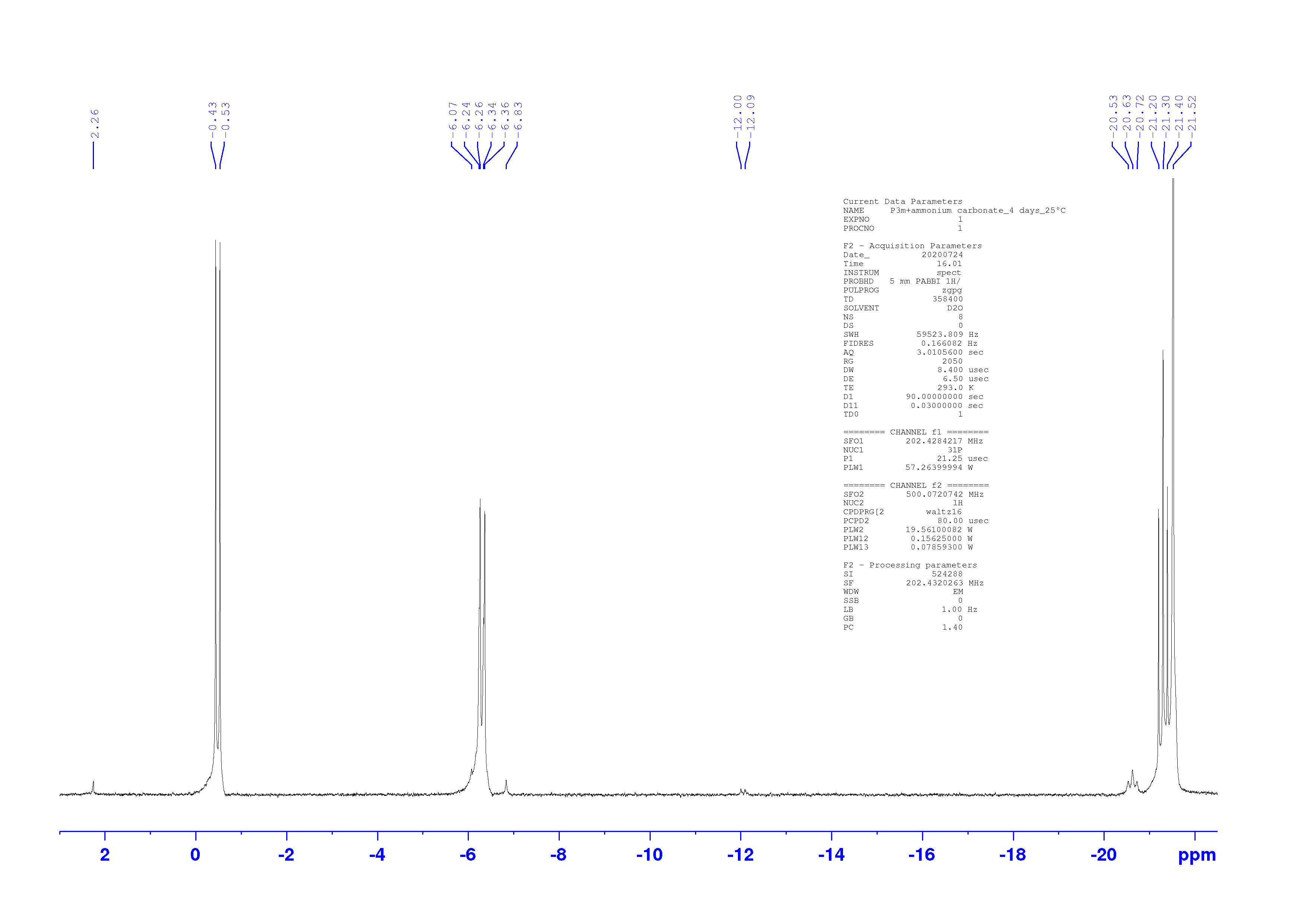


**Fig. S33** ^31^P NMR spectrum of sodium trimetaphosphate with ammonium carbonate in deuterated water after 4 days at 25°C

ATR data:

Sodium trimetaphosphate with ammonium carbonate in water after 4 days at 25°C:

(Bruker, ATR, H_2_O, 25°C, cm^-1^) 1613 (carbonate, C=O sym. stretch.), 1449 (ammonium, –NH bend.), 1358 (carbonate, C=O antisym. stretch. ), 1267 (P3m, O-P-O as. stretch.), 1221 (phosphoramidates, P=O stretch.), 1109 (ammonia, NH_3_ bend.), 1088 (P3m, O-P-O sym. stretch.), 1000 (P3m, P-O-P antisym. stretch.), 924 (phosphoramidates, P-N sym. stretch.), 841 (carbonate, C-O sym. in-plane bending), 774 (P3m, P-O-P sym. stretch.)


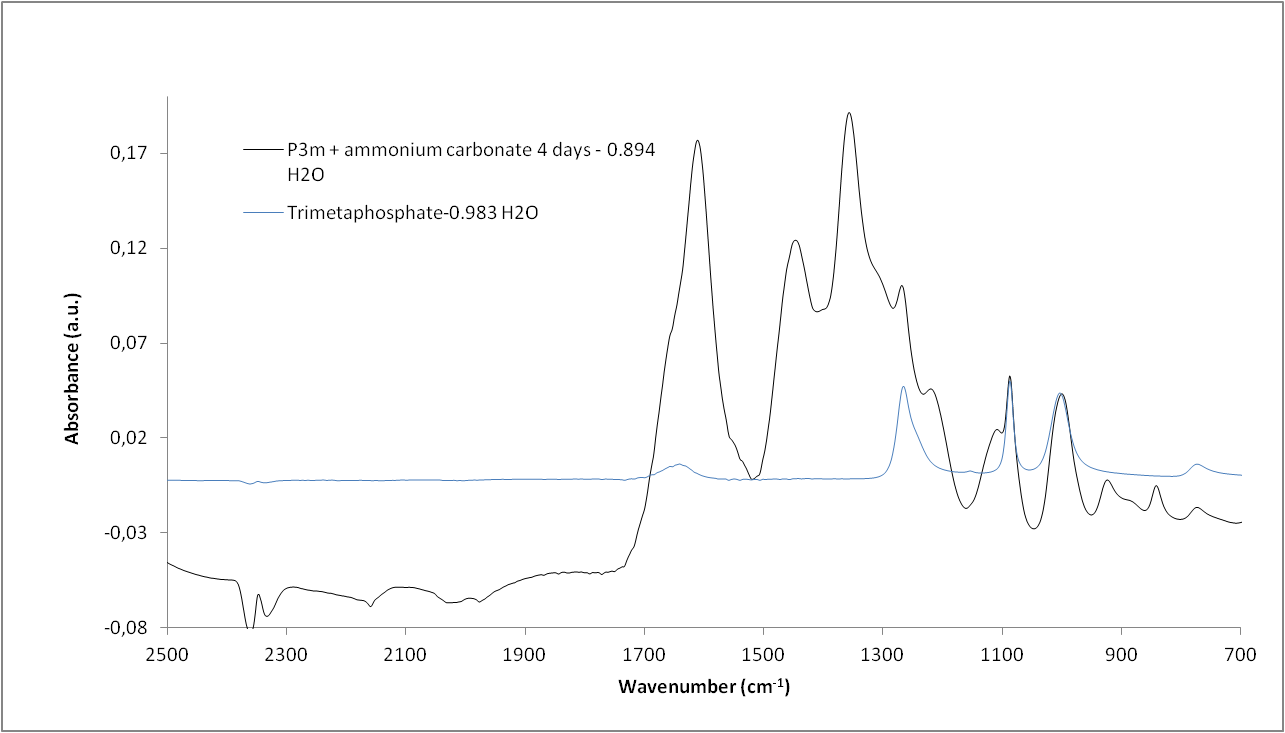


**Fig S34.** ATR-IR spectrum of sodium trimetaphosphate with ammonium carbonate in water after 4 days at 25°C. Water spectrum was removed.

^13^C NMR (Bruker, 125.74 MHz, D_2_O, 25°C, ppm), 17h 12 min acquisition after 4 days ageing at 25°C

Carbamate: δ 166.23 (s); Carbonate: 161.35(s)


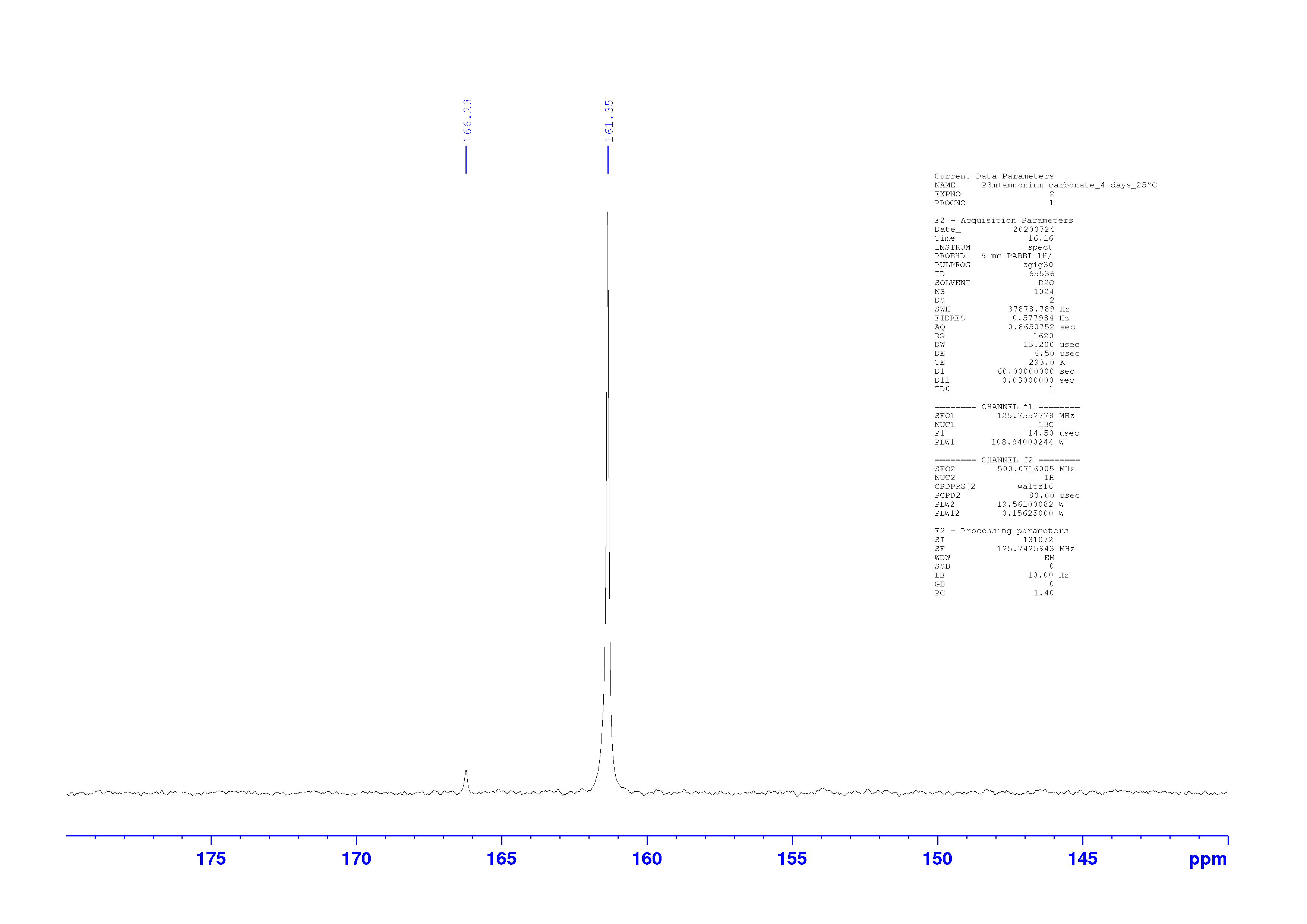


**Fig. S35** ^13^C NMR spectrum of sodium trimetaphosphate with ammonium carbonate in deuterated water after 4 days at 25°C

- 1. Phosphorylation attempts using phosphoramidates

U**rea + phosphoramidates after 70h at 25°C**

NMR data:

^31^P NMR (Bruker, 202.43 MHz, D_2_O, 25°C, ppm), 12 min acquisition after 70h reacting at 25°C

DAP: δ 13.88 (s); MAP: δ 8.24 (s); 1P: δ 3.15 (s); MA3P: δ -0.22 (d, 19.3 Hz), -5.43 (d, 19.3 Hz), -20.70 (t, 19.3 Hz); MA2P: δ -0.62 (d, 20 Hz), -5.68 (d, 20 Hz); 2P: δ -5.74 (s)

^13^C NMR (Bruker, 125.74 MHz, D_2_O, 25°C, ppm)

Urea: δ 163.73 (s)

pH measure: 10.98


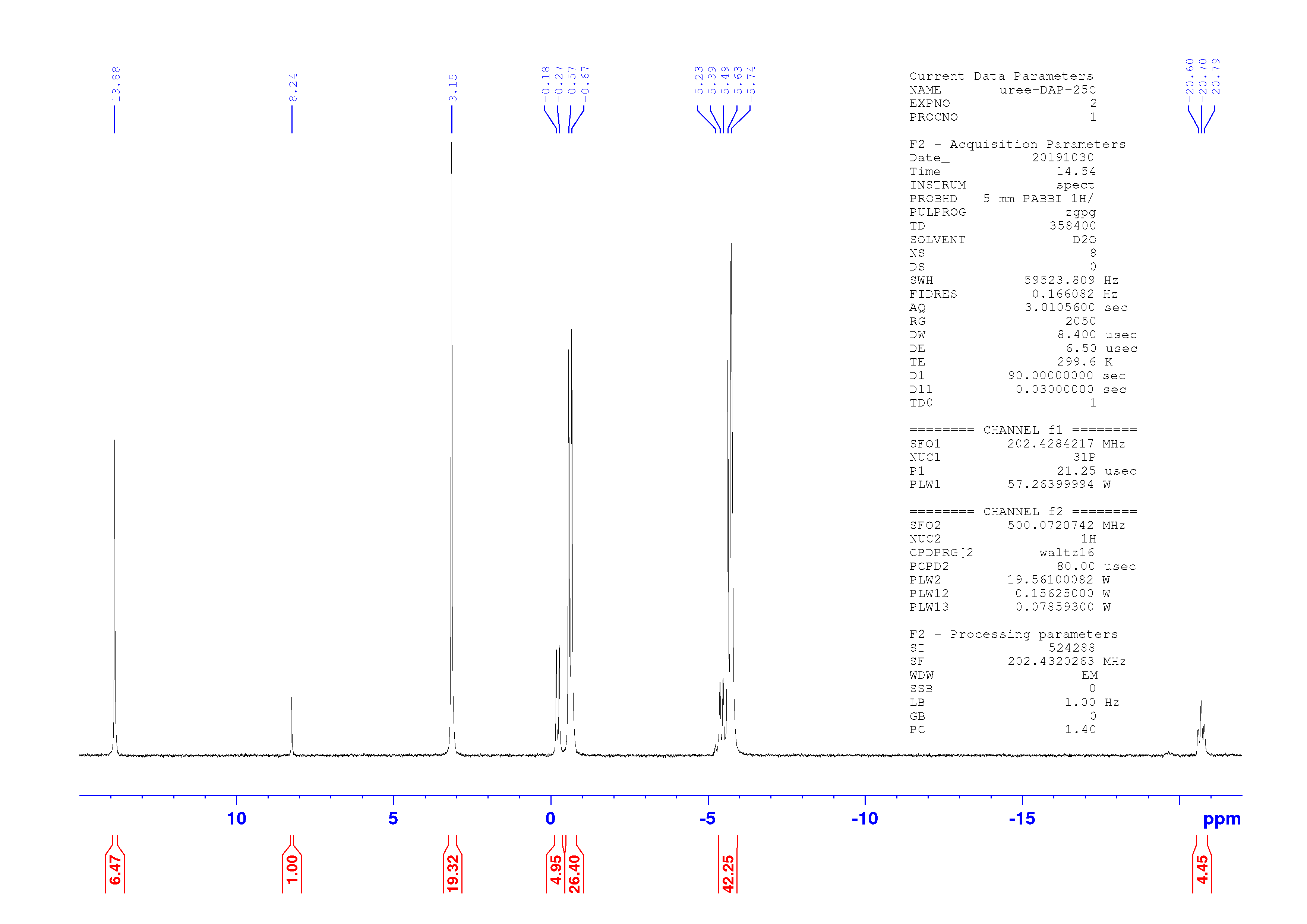


**Fig. S36** ^31^P NMR spectrum of a phosphoramidate solution with urea in deuterated water after 70h at 25°C


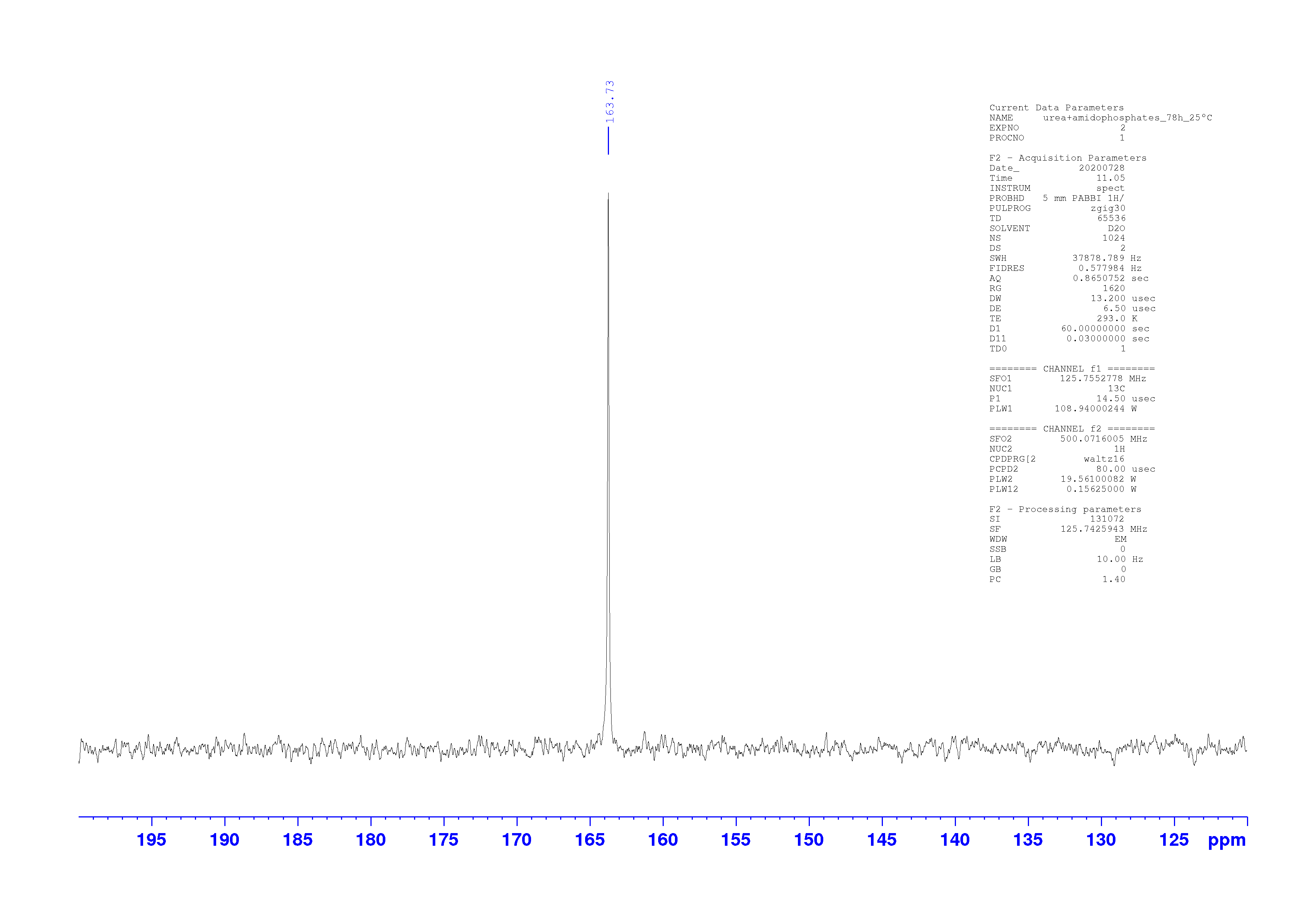


**Fig. S37** ^13^C NMR spectrum of a phosphoramidates solution with urea in deuterated water after 78h at 25°C

ATR data:

Phosphoramidate solution with urea in water after 70h at 25°C:

(Bruker, ATR, H_2_O, 25°C, cm^-1^) 1660 (urea, amide I C=O stretch.), 1628 (urea, NH_2_ antisym. bend.), 1599 (urea, amide II NH_2_ antisym. bending), 1464 (urea, CN antisym. stretch.), 1190 (phosphoramidates, P=O stretch.), 1098/1082/1011(phosphoramidates, P-N/P-O stretch.), 990 (phosphoramidates, P-N stretch.), 932 (phosphoramidates, P-N stretch.), 783 (?)


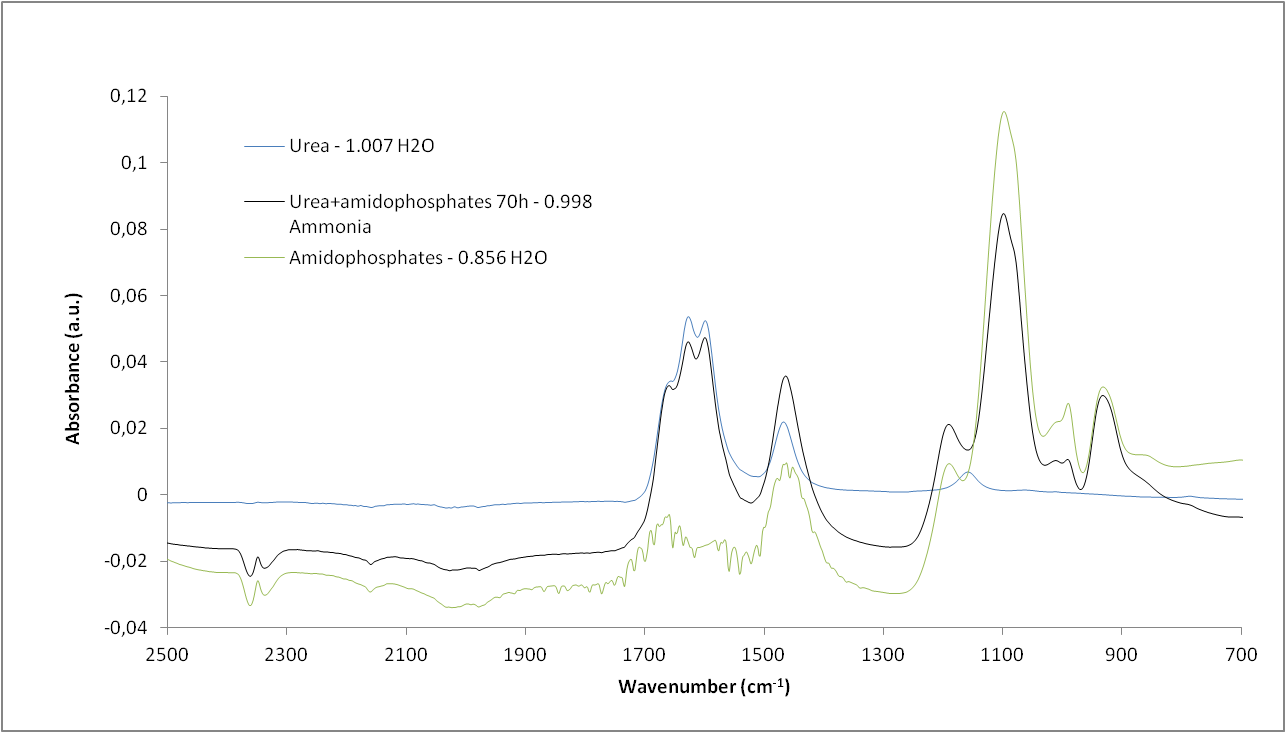


**Fig S38.** ATR-IR spectrum of a phosphoramidate solution with urea in water after 70h at 25°C. Aqueous ammonia spectrum was removed. Comparison with urea and amidophosphates alone.

A**mmonium carbamate + phosphoramidates after 70h at 25°C**

NMR data:

^31^P NMR (Bruker, 202.43 MHz, D_2_O, 25°C, ppm), 12 min acquisition after 70h reacting at 25°C

DAP: δ 13.86 (s); MAP: δ 8.09 (s); 1P: δ 2.94 (s); MA3P: δ -0.29 (d, 19.2 Hz), -5.72 (d, 20.1 Hz), -20.91 (t, 19.6 Hz); MA2P: δ -0.68 (d, 20.0 Hz), -5.87 (d, 20.0 Hz); 2P: δ -6.14 (s)

pH measure: 10.80


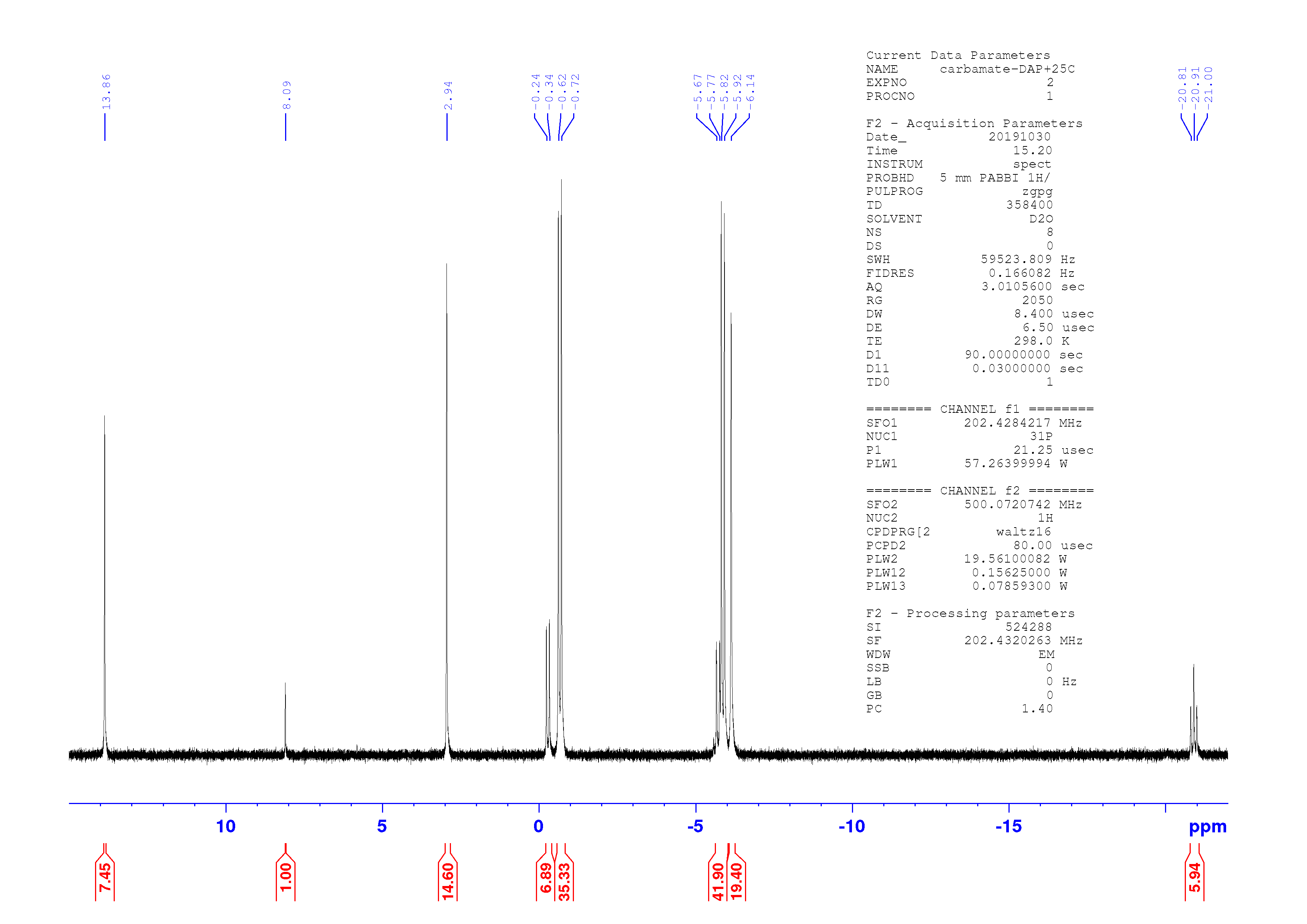


**Fig. S39** ^31^P NMR spectrum of sodium trimetaphosphate with ammonium carbamate in deuterated water after 70h at 25°C

- 1. Empirical table of encountered ^13^P chemical shifts


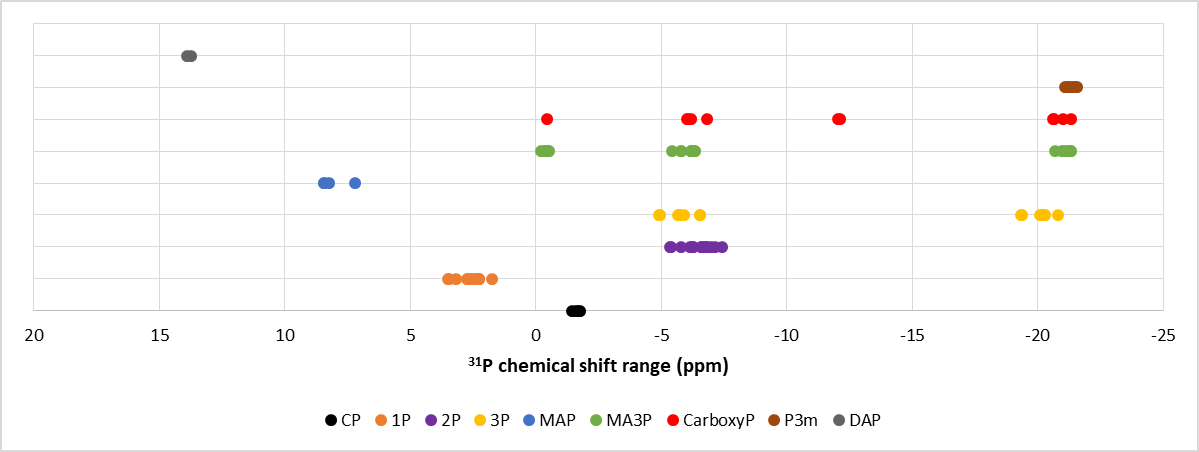


**Fig. S40** Empirical ^31^P chemical shifts of amido- and organophosphate species

This figure lists all ^31^P chemical shifts that have been observed in the present study together with the assignments of the corresponding peaks. The ranges for each compound are mainly due to pH-induced shifts in acido-basic couples.
